# Comorbidity Patterns of Posttraumatic Stress Disorder and Depression Symptoms: Cross-Validation in Two Postearthquake Child and Adolescent Samples

**DOI:** 10.1155/2023/4453663

**Published:** 2023-11-08

**Authors:** Boya Xu, Hao Yuan, Xinchun Wu, Wenchao Wang

**Affiliations:** ^1^Beijing Key Laboratory of Applied Experimental Psychology, National Demonstration Center for Experimental Psychology Education, Faculty of Psychology, Beijing Normal University, Beijing, China; ^2^Pingshan Foreign Languages School, Shenzhen 518118, China; ^3^School of Applied Psychology, Beijing Normal University at Zhuhai, Zhuhai 519087, China

## Abstract

**Background:**

Children and adolescents who have been exposed to a major natural disaster are more likely to suffer from posttraumatic stress disorder (PTSD) and depression. However, only a few studies have examined comorbidity patterns at the symptom level. Furthermore, researchers should validate their findings using multiple samples to address the psychological reproducibility challenge.

**Methods:**

The Child PTSD Symptom Scale (CPSS) and Center for Epidemiological Studies Depression Scale for Children (CES-DC) were administered to two postearthquake child and adolescent samples (Wenchuan earthquake, *N* = 1506; Ya'an earthquake, *N* = 720). Each sample was followed up twice. Comorbidity patterns were characterized by cross-lagged panel network analysis (CLPN), and communities were determined by bootstrap exploratory graphical analysis (bootEGA).

**Results:**

Except for *having difficulty remembering important aspects of the trauma*, the remaining dysphoria symptoms could be considered bridge symptoms between PTSD and depression. Most often, intrusive and avoidant symptoms clustered together, whereas dysphoria symptoms tended to cluster with depressive symptoms. The relationship between PTSD and depression was reciprocal; within PTSD, intrusive symptoms often triggered avoidance symptoms. The correlation coefficient between the two networks was 0.70, and the correlation coefficient of node centrality was 0.55. *Findings*. The association between dysphoria symptoms and depression was strong, and intrusive symptoms constituted the core symptoms of PTSD. Depression and PTSD were causally related, explaining the high comorbidity rates. Two sample networks had similar global characteristics but different local characteristics. The conclusions can be generalized to some extent.

## 1. Introduction

The most common mental disorders among children and adolescents who have experienced a major natural disaster are posttraumatic stress disorder (PTSD) and depression [1]. In children and adolescents who survived an earthquake, the PTSD and depression comorbidity rates range from 6.5% [2] to 39.2% [3]. Why are PTSD and depression so commonly cooccurring? The two may share some common symptoms (e.g., *loss of interest*, *difficulty falling asleep*, and *difficulty concentrating*). Numerous scholars have engaged in relevant explorations; however, the findings remain inconclusive. Some studies have found that upon removing overlapping symptoms, the incidence of comorbidity tends to decrease [4]. Conversely, other studies affirm that even with the elimination of overlapping symptoms, there is no significant alteration in the occurrence of comorbidity [5, 6]. These findings suggest that overlapping symptoms may explain the cooccurrence of PTSD and depression but that other factors may also contribute to the high comorbidity [7].

In addition to overlapping symptoms, some PTSD symptoms are highly correlated with depression. A study by Simms et al. [8] combined the 3 overlapping symptoms mentioned above and 5 nonspecific PTSD symptoms (*difficulty remembering important aspects of the trauma*, *feeling distant or cut off from others*, *emotionally numb*, *future foreshortening*, and *irritable behavior*) as the dysphoria symptoms, which is a representation of negative mood and somatization symptoms shared by PTSD and depression [9]. Armour and Shevlin [10], using confirmatory factor analysis with 12,647 participants, found that the factor loadings for the dysphoria symptoms in PTSD were significantly lower after controlling for depression and generalized anxiety disorder. In another study conducted among soldiers, the dysphoria symptom was more strongly correlated with depression than with other PTSD symptoms [11]. Therefore, the dysphoria symptoms may also contribute to high comorbidity.

More importantly, another study found a reciprocal relationship between PTSD and depression via a longitudinal design [12], providing another explanation for their comorbidity. However, a definitive consensus regarding the direction between PTSD and depression has not yet been reached. Some studies have found that depression can predict subsequent PTSD [13–15] but that PTSD cannot predict depression [15]. However, another study found that PTSD can predict depression but that depression cannot predict PTSD [4]. The reciprocal relationships between PTSD and depression require further elucidation.

Most of the studies cited above are based on the common factor hypothesis, which views symptoms as representations of disorders and uses latent variables to explain the covariance among symptoms. The network theory of mental disorders, however, suggests that explaining symptom covariation with latent variables is unnecessary since there are reciprocal relationships between them. A complex system should be used to describe the reciprocal relationships between symptoms, referred to as a symptom network. A network is composed of symptoms, also known as nodes. The comorbidity of different mental disorders can be defined as the relationship between nodes belonging to different networks, and the nodes connecting two networks are known as bridge nodes [16]. In recent years, researchers have tended to adopt network theory for studying mental disorders and to conduct data fitting through network analysis models. Unlike latent variable models guided by the common factor hypothesis, network analysis models do not require satisfying the local independence assumption [17] and are able to accommodate feedback loops between symptoms, and the relative importance of symptoms varies and is not “equally” important or “interchangeable,” which better represents clinical practice [18, 19].

Researchers have begun to use network analysis models to construct PTSD and depression comorbidity network. Overlapping symptoms and some dysphoria symptoms have been identified as bridge symptoms [20–22]. For example, according to Duek et al. [23], the three most influential nodes in the comorbidity network were *feeling distant or cut off from others*, *difficulty concentrating*, and *diminished interest*. Moreover, in most studies, overlapping symptoms are not only a central component of the comorbidity network but also the core bridging symptoms between PTSD and depression [24–27].

In conclusion, first, scholars have often concentrated their focus on overlapping symptoms, overlooking the significance of dysphoria symptoms. Only one study involved children and adolescents; however, dysphoria symptoms were not included [20]. Second, most of these studies were conducted with participants who had suffered human-caused traumatic events; none were conducted with survivors of natural disasters. It is well known that the type of trauma influences the PTSD symptom network [28], thus potentially having implications on the PTSD and depression comorbidity network. Third, based on cross-sectional data, only Lazarov et al. [29] constructed a Bayesian directed network, but this network does not allow feedback between symptoms. The network theory suggests that there are reciprocal relationships between symptoms and that feedback loops may form on this basis. Fourth, only Duek et al. [23] used a large sample to cross-validate their findings, while Yarkon and Westfall [30] suggested that cross-validating results among different samples may be more effective.

In this study, two follow-up surveys were conducted with children and adolescents from the areas affected by the Wenchuan earthquake (12 May 2008, magnitude 8.0 on the Richter scale) and the Ya'an earthquake (20 April 2013, magnitude 7.0 on the Richter scale). A directed PTSD and depression comorbidity network was constructed using a cross-lagged network analysis model, and the importance of dysphoria symptoms in comorbidity network was explored. We also cross-validated the results between the two samples to increase the credibility of our findings.

## 2. Methods

### 2.1. Procedure and Participants

One year after the Wenchuan earthquake (May 2009, T1), 2,577 students in Wenchuan and Mao counties were surveyed. One and a half years after the Wenchuan earthquake (November 2009, T2), 1,506 (58.4%) students surveyed in T1 were followed up. Among them, there were 699 (46.4%) boys. At T1, the average age of them was 13.08 (SD = 2.19) years, ranging from 9 to 18 years. One year after the Ya'an earthquake (May 2014, T1), 889 students in Lushan County were surveyed. One and a half years after the Ya'an earthquake (November 2014, T2), 720 (80.9%) students surveyed in T1 were followed up. Among them, there were 326 (45.3%) boys. At T1, the average age of them was 13.82 (SD = 2.75) years, ranging from 9 to 18 years; 29 students did not provide their age.

Current clinical and counselling psychology students administered all self-report questionnaires in class groups and collected all data on the spot. After the questionnaires were administered, students were counselled in groups to alleviate any discomfort that might have been caused. All procedures were approved by the students, their parents, the principal, and the local education bureau as well as by the ethics committee of our institution.

### 2.2. Measures

The Child PTSD Symptom Scale for *DSM-IV* (CPSS; [31]) was used to assess PTSD symptoms. The CPSS consists of 17 items on a 4-point scale from 0 (*never*) to 3 (*always*). It has good reliability and validity among postearthquake children and adolescents [32]. For the Wenchuan sample, Cronbach's *α* coefficients were 0.87 (T1) and 0.89 (T2), and for the Ya'an sample, they were 0.87 (T1) and 0.90 (T2). As recommended by Foa et al. [31], 11 was used as the cut-off score to screen for PTSD symptoms in children.

The Center for Epidemiologic Studies Depression Scale for Children (CES-DC; [33]) was used to assess depression symptoms. The CES-DC consists of 20 items scored on a 4-point scale from 0 (*never*) to 3 (*always*) and includes four reverse scoring questions. It has good reliability and validity among postearthquake children and adolescents [32]. For the Wenchuan sample, Cronbach's *α* coefficients were 0.87 (T1) and 0.89 (T2), and for the Ya'an sample, they were 0.90 (T1 and T2). As recommended by Weissman et al. [34], 15 was used as the cut-off score for screening for depression symptoms in children and adolescents.

### 2.3. Data Analysis

#### 2.3.1. Missing Data

The percentage of missing data in the Wenchuan sample was 1.3% at T1 and 0.9% at T2; for the Ya'an sample, the percentage of missing data was 0.3% at T1 and 0.4% at T2. We used *mice* function in the R package mice [35] for missing data, as suggested by Levinson et al. [36].

#### 2.3.2. Network Estimation and Visualization

A cross-lagged panel network model (CLPN) was constructed to examine temporal relationships between symptoms [37]. The CLPN combines the strengths of the cross-lagged model and network analysis models, and it is consistent with the network theory assumption that symptoms are reciprocally related [38, 39]. The CLPN was estimated using a series of regression to compute autoregressive (that is, the coefficient for a symptom at T1 predicting itself at T2 after controlling for all other symptoms at T1) and cross-lagged (that is, the coefficient for a symptom at T1 predicting a different symptom at T2 after controlling for all other symptoms at T1) models. A 10-fold cross-validation tuning parameter selection method was used to reduce false-positive edges by regularizing the regression coefficients using LASSO. These steps were accomplished using the *cv.glmnet* function of the R package *glmnet*.

The CLPN was visualized using the Fruchterman-Reingold algorithm [40]. Stronger connections are represented by thicker and more saturated edges, and nodes with more and/or stronger connections are positioned closer together. The averageLayout function of the R package *qgraph* was used to create an identical layout of nodes according to their average position across networks to facilitate visual comparison. For both networks, the minimum edge value was 0. The maximum edge value was 0.21 (absolute value), which is the maximum edge of the two samples (excluding autoregressive edges).

#### 2.3.3. Centrality Estimation

We used the bridge-expected influence index (BEI) to identify the bridge symptoms between PTSD and depression in accordance with Jones et al. [41]. The BEI of a node is derived from its EI. To calculate the EI of a node, all edges associated with that node are added together. The calculation of BEI is similar to that of EI; when calculating the BEI of a node, only the edges between that node and the nodes of other disorders are considered, and the edges between that node and the nodes of which the disorder that node itself is located are not considered.

#### 2.3.4. Accuracy and Stability Estimation

In accordance with the standard network analysis procedures, we examined the accuracy and stability of the network using two bootstrap methods implemented in the R package *bootnet* [42]. First, we estimated the accuracy of the edge weights by calculating 95% confidence intervals (CIs) around each edge using a nonparametric bootstrap of 1,000 instances. Second, we used casedrop bootstrapping to estimate the correlation stability coefficients to determine BEI rank-order stability [42]. The CS coefficient should be greater than 0.25, preferably greater than 0.50. Third, we determined whether the BEI difference between nodes and edge weights between edges was significantly nonzero (*α* = 0.05).

#### 2.3.5. Identifying Communities

To identify communities within the PTSD and depression comorbidity networks, we used exploratory graphical analysis (EGA). EGA, recently proposed in the context of network psychometrics, is a new method of estimating the number of factors driving multivariate data. EGA has been shown to outperform many traditional methods [43]. To ensure the accuracy of EGA, Christensen and Golino [44] recommended performing bootstrap exploratory graph analysis (bootEGA). The sampling procedure was repeated 1000 times, followed by an intercept of the median network for reporting. It is important to note that bootEGA cannot be applied to the CLPN since the CLPN is a directed network and bootEGA is only applicable to undirected networks. Therefore, we used two samples of cross-sectional data for bootEGA.

### 2.4. Network Replicability

Following the suggestion of Funkhouser et al. [39], we investigated the degree of similarity between two CLPNs in four ways: (a) correlation between edge lists, (b) number of edge instances replicated across CLPNs (both by sign and by presence), (c) correlations of centrality indices between networks, and (d) consistency in the most central symptoms.

## 3. Results

### 3.1. Descriptive Statistics

The demographics and trauma exposure of the two samples are presented in Table 1, and the prevalence of PTSD and depression is shown in Table A1. The labels and abbreviations for each CPSS and CES-DC item are presented in Table A2, and the descriptive statistics for each item are presented in Table A3 and Table A4. Based on the standard procedures for network analysis [42], although none of the symptoms in this study violated a normal distribution (skewness > 2 or kurtosis > 7; [45]), the *huge* function in the R package huge was used to transform the data before network analysis.

### 3.2. Network Estimation and Accuracy Testing

**Figure 1:**
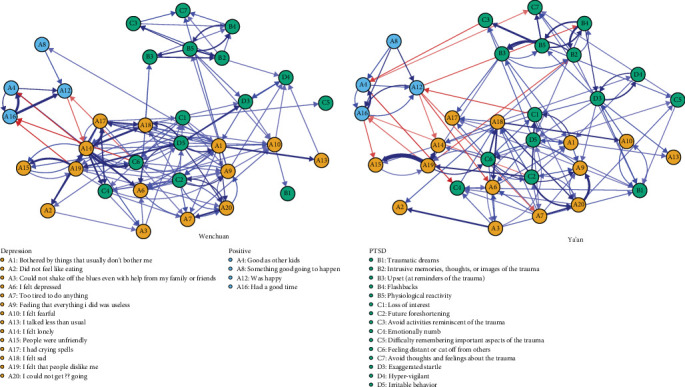
The cross-lagged panel networks for two samples. Note. Arrows represent unique longitudinal relationships. Blue edges indicate positive relationships, and red edges indicate negative relationships. Edge thickness represents the strength of the cross-lagged coefficients such that thicker edges represent stronger relations. Autoregressive edges and weaker edges (i.e., cross-lagged coefficients within 0 ± 0.05) were excluded from the plot to ease visual interpretation.

Figure 1 illustrates the comorbidity network of PTSD and depression in the Wenchuan and Ya'an samples. In the network, the arrows represent temporal pairwise relationships between symptoms when controlling for all other symptoms at T1 (edge weights are presented in Table A5 and Table A6, and all autoregressive edges are presented in Figure A1). Autoregressive edges (mean edge weight = 0.19) were substantially stronger than cross-lagged edges (mean edge weight = 0.01), and the plotting algorithm determined the edge thickness relative to the strongest edge; therefore, the autoregressive edges were excluded from Figure 1 (for the sake of visualization, only edges with an absolute value greater than 0.05 are presented in Figure 1) to make the cross-lagged edges more visually interpretable. As seen in Figure 1, the node clustering pattern was different from that for the *DSM-IV*. Some symptoms of PTSD (B2 to B5, C3, and C7) clustered together, and the other nodes of PTSD (C2, C4, C6, D5, and C1) were visually closer to depression symptoms; node D5 is located at the hub of the network.

The global characteristics of each network are shown in Table 2. It can be seen that the Wenchuan sample has more nonzero edges than the Ya'an sample and that the average edge weights of the two samples are approximately equal; therefore, the Wenchuan sample has greater global strength. The density within each community was greater than between communities; there were more nonzero edges from depression to PTSD than from PTSD to depression in the Wenchuan sample, as with total weights. In the Ya'an sample, there were significantly more edges from PTSD to depression than from depression to PTSD, and the difference in total weight was larger. For the nonzero edges between PTSD and depression, dysphoria symptoms accounted for a greater proportion of strength, approximately 75%. As shown in Table A7, dysphoria symptoms C2/C6/D5 were all ranked in the top three in at least one sample. In each sample, at least 4 dysphoria symptoms were ranked in the top 10; only C5 was ranked outside the top 15. The top 5 weighted edges of each network are shown in Table 3. Within the PTSD community, the edges with higher weights were mostly between intrusive symptoms, and it is more likely that intrusive symptoms triggered avoidant symptoms (see Table A5 to Table A6 and Figure 1). Within the depression community, the edges with higher weights were mostly between damaged relationships (nodes A14, A16, and A19) and sadness/isolate (nodes A17 and A15). For edges from PTSD to depression, the edges with higher weights were emitted mainly by nodes D5 and C2 and point to nodes A19, A18, A9, and A17 of depression. As for edges from depression to PTSD, the edges with higher weights mostly originated from depression nodes (A14, A19, and A9) and point to nodes C6, C4, and C2 of PTSD. The 95% CIs for each edge in the two networks are shown in Figure A2. Even though the 95% CIs for each edge had a wide range and must be interpreted carefully, the 95% CIs for edges with larger weights did not overlap with edges with smaller weights.

**Figure 2:**
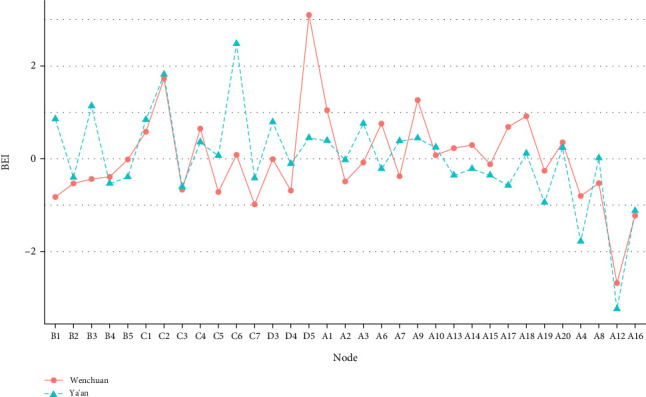
The normalized BEI of nodes for each sample. Note. B1: traumatic dreams; B2: intrusive memories, thoughts, or images of the trauma; B3: upset (at reminders of the trauma); B4: flashbacks; B5: physiological reactivity; C1: loss of interest; C2: future foreshortening; C3: avoid activities reminiscent of the trauma; C4: emotionally numb; C5: difficulty remembering important aspects of the trauma; C6: feeling distant or cut off from others; C7: avoid thoughts and feelings about the trauma; D3: exaggerated startle; D4: hypervigilant; D5: irritable behavior; A1: bothered by things that usually do not bother me; A2: did not feel like eating; A3: could not shake off the blues even with the help from my family or friends; A6: I felt depressed; A7: too tired to do anything; A9: feeling that everything I did was useless; A10: I felt fearful; A13: I talked less than usual; A14: I felt lonely; A15: people were unfriendly; A17: I had crying spells; A18: I felt sad; A19: I felt that people dislike me; A20: I could not get “going”; A4: good as other kids; A8: something good going to happen; A12: was happy; A16: had a good time.

In Figure 2, the normalized BEIs for each sample are shown; it can be seen that the number of bridge nodes across samples is not completely uniform, but some commonalities still exist. Node C2 (dysphoria symptom) is considered a bridge node since its normalized BEI values in both samples are greater than 1. In the Wenchuan sample, the nodes with the highest BEI were D5 (3.10) and C2 (1.73); for the Ya'an sample, the nodes with the highest BEI were C6 (2.47) and C2 (1.81).

The BEI CS coefficients were 0.59 for Wenchuan and 0.28 for Ya'an, and the stability pattern can be seen in Figure A3. The BEI CS coefficients were greater than 0.25 for both samples and even greater than 0.5 for the Wenchuan. In Figure A4, bootstrapped BEI difference test results for the two samples are shown; nodes with a larger BEI have a statistically significantly larger BEI than do other nodes in the network. The bootstrapped difference tests between edge weights of the two samples are shown in Figure A5.

### 3.3. Community Identification

The communities in the PTSD and depression comorbidity network were identified using bootEGA (Table 4). Although the community classification patterns were not identical across samples at each time point, there were still some commonalities among them. In most cases, dysphoria symptoms did not form a community with the other PTSD symptoms and were more likely to be combined with depression in some cases. The 9 nondysphoria PTSD symptoms formed a separate community in most cases. Specifically, intrusive symptoms (B1 to B5), avoidant symptoms (C3 and C7), and fear (A10) were clustered into a community in most cases; damaged relationships (A14/A15/A19), crying (A17), and sadness (A18) in depression were clustered as a community in all cases; the four positive emotions (A4/A8/A12/A16) were clustered into a community in most cases; and the dysphoria symptoms were more likely to form a community with the remaining depressive symptoms.

### 3.4. Network Replicability

According to Table 5, first, the correlation coefficient between the edge lists was 0.70, i.e., higher than 0.61 reported by Funkhouser et al. [39]. Second, a total of 288 edge instances were replicated across two samples (both by sign and by presence), representing 61.8% of the edges in the Ya'an network and 47.4% in the Wenchuan. In this respect, there was a degree of similarity in the comorbidity network of PTSD and depression between the two postearthquake child and adolescent samples. In addition, it is important to note that the BEI correlation coefficient was not very high (0.55) and that the bridge symptoms showed some minor differences across the two samples, as shown in Figure 2. As a result, we concluded that the comorbidity networks of PTSD and depression in the Wenchuan and Ya'an samples were consistent in global characteristics but were different with regard to local characteristics.

## 4. Discussion

This study followed two postearthquake child and adolescent samples, modelled them separately using the CLPN, identified PTSD and depression comorbidity patterns at the symptom level, confirmed the role of dysphoria symptoms as bridging symptoms between PTSD and depression, improved conclusion generalizability by comparing the findings between two samples, and provided theoretical support for clinical application.

### 4.1. Dysphoria Symptoms

The proportion (75%) of dysphoria symptoms occupying non-zero-weighted edges linking PTSD and depression underscores the significance, suggesting that dysphoria symptoms play an influential role in the comorbidity network. Except for node C5, all remaining dysphoria symptoms can be viewed as bridge nodes between PTSD and depression, a finding that is consistent with the results reported by Afzali et al. [24], Choi et al. [46], Price et al. [47], and Gilbar [26]. Node C5 is classified as a peripheral symptom in the network (Figure 1), which is consistent with previous network analyses of PTSD alone*. Difficulty recalling important aspects of trauma* tends to have the lowest centrality index among all PTSD symptoms [29].

Furthermore, the bootEGA results were consistent with the findings reported by Gros et al. [48], who used factor analysis to explore the structure of PTSD and depression. On the one hand, this indicates that the relationship between dysphoria symptoms and depression is stronger than the 9 specific PTSD symptoms; on the other hand, it also suggests that *DSM-IV* boundaries are inaccurate. The *ICD-11*, which was released in 2018, includes only six specific symptoms of PTSD; dysphoria symptoms are not included [49]. This suggests that at least in the population of children and adolescents who have experienced a major natural disaster, the *DSM-IV* symptom boundaries may not be applicable when considering the PTSD and depression comorbid network structure.

### 4.2. The Reciprocal Effect of PTSD and Depression

From a macroscopic perspective (Table 2), it can be observed that the relationship between PTSD and depression was mutually reinforcing. Based on the nonzero edge total weights, the impact of the two on each other was almost equal in the Wenchuan sample. Nevertheless, for the Ya'an sample, the effect of PTSD on depression was stronger than the effect of depression on PTSD. In line with network theory, it follows that when a symptom of PTSD or depression is activated after a traumatic event, it will propagate through the comorbid network based on the causal links and bridge symptoms, leading to the activation of the entire comorbid network. This also explains the high comorbidity of PTSD and depression and is consistent with the results of earlier studies [14, 50, 51]. However, these studies used latent variable models to study the correlation between PTSD and depression at the disorder or dimensional level rather than at the symptom level.

Within the PTSD community, intrusive symptoms are more likely to trigger avoidance symptoms, which is consistent with findings reported by Lazarov et al. [29]. PTSD stress response theory [52] posits that after a traumatic event, if an individual fails to assimilate the negative cognition brought about by the event into his or her cognitive schema, memories and cues of the event will continue to lodge in the individual's mind (e.g., intrusive memories, thoughts, or images of the trauma/flashbacks), and then, the individual will have physical and psychological reactions (e.g., upset at reminders of the trauma/physiological reactivity). When this occurs, the individual will take defensive avoidance measures to counteract these negative physiological and psychological reactions. In the depression community, the edges with higher weights were mainly between damaged relationships (nodes A14, A16, and A19) and sadness/isolation (nodes A17 and A15), a result that is consistent with the findings reported by An et al. [25]. These findings suggest that these symptoms may be the most important indicators of depression.

### 4.3. Cross-Validation

In the present study, the PTSD and depression comorbidity network structure in the two samples was cross-validated, and similar global characteristics with a few differences at the local level were found. The following are a few possible reasons for these differences. First, the Wenchuan earthquake had a larger magnitude, causing greater damage and reaching a broader spectrum of individuals and regions, and postearthquake rescue was very difficult due to the geographical location of Wenchuan. The Chinese government gained a great deal of experience responding to natural disasters and improving its infrastructure and rescue capabilities after the Wenchuan earthquake. The accumulated experiences from the Wenchuan earthquake were applied to the rescue efforts after the Ya'an earthquake, mitigating a portion of the adverse consequences following the Ya'an earthquake. Second, the ethnic composition of the samples differed, with Chinese comprising almost all of the Ya'an sample (Table A1); Han Chinese accounted for only 17.5% of the Wenchuan sample. The Wenchuan earthquake occurred in Aba (Ngawa) Tibetan and Qiang Autonomous Prefecture, Sichuan Province, China, where Tibetan and Qiang occupy a greater area and have customs and religious beliefs that are different from those of Han Chinese people. Third, the sample size is different; Wenchuan is twice as large as Ya'an, and this difference might affect the network structure [53]. Fourth, there were significant differences in participant grade levels between the two samples. The participants in the Wenchuan sample were mostly primary school students, and the participants in the Ya'an sample were predominantly middle school students. Primary school students typically lack mature coping mechanisms, making them more vulnerable to substantial impacts in the face of significant natural disasters. Fifth, the degree of trauma exposure varied. In the Wenchuan sample, survivors had higher levels of trauma exposure. The number of survivors who were trapped after the earthquake was 19.5%, and the survivors who experienced the death of relatives and friends because of the earthquake were even higher, i.e., 36.3%. In contrast, in the Ya'an sample, 8.1% of the survivors were trapped after the earthquake, and 12.6% of the survivors experienced the death of relatives and friends during the earthquake. These differences may exert multifaceted influences on the postdisaster psychological well-being of individuals.

### 4.4. Strengths and Limitations

The following strengths of this study should be emphasized. First, to the best of our knowledge, this is the first study to construct a PTSD and depression comorbidity network based on longitudinal data for children and adolescents who have experienced a major natural disaster, revealing bridge symptoms between them and elucidating the comorbidity pattern. When assessing the mental health status of children and adolescents after a major natural disaster, clinical workers should consider the cooccurrence of PTSD and depression, identify bridge symptoms to screen high-risk students, and use these symptoms as a catalyst for developing intervention strategies. Second, we used the CLPN to model longitudinal data for the two samples and concluded that there is a reciprocal effect of PTSD and depression at the symptom level. Third, in this study, we identified communities in the PTSD and depression comorbidity network using bootEGA. Taking a closer look at transcending traditional diagnostic categories can enhance our understanding of the comorbidity of PTSD and depression. Finally, this study was cross-validated using two independent samples, responding to the challenge of reproducibility in psychology and enhancing the generalizability of our results.

It is important to note that this study has some limitations. First, as mentioned above, there was some heterogeneity between the two samples, which is one of the reasons for the differences in local characteristics between the two networks. However, the correlation between edge lists was 0.70, which indicates that the results of this study are also generalizable across samples. Future studies should consider both the heterogeneity and homogeneity of the samples as well as the generalizability and specificity of the results. Second, all samples were nonclinical and used self-report methods to measure PTSD and depression, an approach that may have led to an overestimation of the severity of PTSD and depressive symptoms. The results of this study can be compared and validated with clinical samples to further the understanding of PTSD and depression comorbidity. Third, similar to other multivariate statistical analysis methods, the network in this study did not include external field variables that could have affected the PTSD and depression comorbidity patterns. Finally, three new items related to negative cognition and mood added to the *DSM-5* for PTSD were not included in the analysis. In future research, *DSM-5* criteria should be used to construct a PTSD and depression comorbidity network that can be compared with that developed in this study.

## 5. Conclusion

In this study, longitudinal data were used to construct cross-lagged panel network models in two postearthquake samples of children and adolescents to investigate PTSD and depression comorbidity patterns at the symptom level and cross-validate the results. In addition to difficulty remembering important aspects of trauma symptoms, the remaining 7 dysphoria symptoms were bridge symptoms; intrusive and avoidant symptoms tended to cluster as a community and were specific to PTSD; the relationship between dysphoria symptoms and depression was much stronger, and these two were more likely to come together as a community. In the PTSD community, intrusive symptoms were more likely to precede avoidant symptoms. There were some similarities between the two networks regarding global characteristics and some differences regarding local characteristics, and our findings have some generalizability.

## Figures and Tables

**Table 1:** Demographic information and trauma exposure of samples.

Variables	Wenchuan		Ya'an	
	Frequency	%	Frequency	%
*Ethnic*				
Han	264	17.5	708	98.3
Tibetan	389	25.8	9	1.3
Qiang	805	53.5	—	—
Others	46	3.1	—	—
*Grade (T1)*				
Primary 4	388	25.8	136	18.9
Primary 5	289	19.2	146	20.3
Primary 6	26	1.7	—	—
Grade 7	249	16.5	107	14.9
Grade 8	322	21.4	134	18.6
Grade 10	63	4.2	83	11.5
Grade 11	165	11.0	112	15.6
*Trauma exposure*				
Trapped in the earthquake	293	19.5	58	8.1
Injured in the earthquake	92	6.1	99	13.8
Father/mother died in the earthquake	127	8.4	14	1.9
Teacher died in the earthquake	136	9.0	3	0.4
Classmate died in the earthquake	457	30.3	123	17.1
Relatives/friends died in the earthquake	546	36.3	91	12.6
Houses were severely or completely damaged in the earthquake	970	64.4	488	67.8
Fear of injury in the earthquake	948	62.9	452	62.8
Fear of death in the earthquake	889	59.0	442	61.4

*Note.* Some variables have missing values and therefore do not reach the total sample size.

**Table 2:** Global characteristics of each network.

		Wenchuan	Ya'an
All estimated edges	The entire network	1089	1089
	PTSD community	225	225
	Depression community	196	196
	Edges from PTSD to depression	210	210
	Edges from PTSD to depression	210	210

Edges with nonzero weights	The entire network	586	450
	PTSD community	141	121
	Depression community	127	89
	Edges from PTSD to depression	104	99
	Edges from PTSD to depression	109	63

Network density	The entire network	0.54	0.41
	PTSD community	0.63	0.54
	Depression community	0.65	0.45
	Edges from PTSD to depression	0.50	0.47
	Edges from PTSD to depression	0.52	0.30

Global strength	The entire network	23.54	23.08
	PTSD community	6.59	7.76
	Depression community	6.70	6.16
	Edges from PTSD to depression	3.42	3.46
	Edges from PTSD to depression	3.44	2.19

Average edge weights	The entire network	0.02	0.02
	PTSD community	0.03	0.03
	Depression community	0.03	0.03
	Edges from PTSD to depression	0.02	0.02
	Edges from PTSD to depression	0.02	0.01

**Table 3:** Top 5 weighted edges of each network.

	Wenchuan		Ya'an	
	Edge	Weight [95% CI]	Edge	Weight [95% CI]
PTSD community	B5⟶B3	0.12 [0.06~0.18]	B5⟶B3	0.14 [0.06~0.23]
	B3⟶B2	0.11 [0.05~0.16]	B4⟶B2	0.13 [0.04~0.22]
	D5⟶D3	0.10 [0.04~0.17]	B5⟶C3	0.13 [0.04~0.21]
	B5⟶B2	0.10 [0.04~0.16]	B4⟶B5	0.11 [0.02~0.21]
	B4⟶B5	0.10 [0.04~0.16]	B5⟶B4	0.11 [0.01~0.20]

Depression community	A14⟶A17	0.15 [0.09~0.22]	A19⟶A15	0.20 [0.11~0.29]
	A16⟶A12	0.14 [0.07~0.21]	A19⟶A14	0.20 [0.10~0.30]
	A16⟶A4	0.13 [0.08~0.19]	A20⟶A9	0.14 [0.06~0.22]
	A14⟶A18	0.13 [0.06~0.19]	A16⟶A4	0.12 [0.03~0.21]
	A19⟶A15	0.12 [0.06~0.19]	A3⟶A2	0.12 [0.01~0.23]

PTSD to depression	D5⟶A19	0.12 [0.06~0.17]	C2⟶A19	0.11 [0.04~0.19]
	D5⟶A1	0.11 [0.05~0.16]	C2⟶A20	0.11 [0.03~0.19]
	D5⟶A18	0.10 [0.04~0.16]	C6⟶A18	0.11 [0.02~0.20]
	D5⟶A17	0.09 [0.04~0.15]	C6⟶A9	0.09 [0.00~0.18]
	D5⟶A6	0.09 [0.03~0.15]	C2⟶A9	0.09 [-0.00~0.18]

Depression to PTSD	A14⟶C6	0.15 [0.08~0.21]	A19⟶C6	0.09 [-0.00~0.19]
	A14⟶C4	0.11 [0.05~0.17]	A1⟶C1	0.09 [0.00~0.18]
	A17⟶C4	0.10 [0.04~0.16]	A9⟶C6	0.09 [-0.00~0.19]
	A20⟶C2	0.10 [0.04~0.16]	A10⟶D3	0.09 [-0.00~0.18]
	A9⟶C2	0.08 [0.02~0.13]	A10⟶B1	0.09 [-0.00~0.19]

*Note.* Except for the autoregressive edges.

**Table 4:** Community classification patterns for each samples.

Node name	Wenchuan		Ya'an	
	T1	T2	T1	T2
B1	3	3	2	2
B2	3	3	2	2
B3	3	3	2	2
B4	3	3	2	2
B5	3	3	2	2
C1	2	2	1	1
C2	1	1	1	1
C3	3	3	2	2
C4	2	2	1	1
C5	2	2	1	2
C6	1	1	1	1
C7	3	3	2	2
D3	2	2	2	2
D4	2	2	2	2
D5	1	2	1	1
A1	1	1	1	1
A2	2	2	1	1
A3	1	1	1	1
A6	1	1	1	1
A7	1	1	1	1
A9	1	1	1	1
A10	2	2	2	2
A13	6	1	1	1
A14	4	4	3	3
A15	4	4	3	3
A17	4	4	3	3
A18	4	4	3	3
A19	4	4	3	3
A20	1	1	1	1
A4	5	5	4	4
A8	6	6	4	4
A12	5	5	4	4
A16	5	5	4	4

*Note.* The same number in each column means that these nodes are in the same community. Dysphoria symptoms: C1: loss of interest; C2: future foreshortening; C4: emotionally numb; C5: difficulty remembering important aspects of the trauma; C6: feeling distant or cut off from others; D5: irritable behavior.

**Table 5:** Replicability of the network in the two samples.

	Wenchuan and Ya'an		
	*r*	95% CI	*t*
Pearson correlation coefficient of edge lists	0.70⁣^∗∗∗^	[0.68~0.74]	33.24
Pearson correlation coefficient of BEI	0.55	[0.25~0.75]	3.66
Number of a specific edge replicated	288 (26.45%)		

*Note. *⁣^∗^*p* < .05, ⁣^∗∗^*p* < .01, and ⁣^∗∗∗^*p* < .001.

## Data Availability

Where necessary, the data sets used and analyzed in this study are available from the authors.

## Supplementary Materials

Supplementary 1 Table A1: prevalence and comorbidity of PTSD and depression in two samples. Table A2: the labels and abbreviations for each CPSS and CES-DC item. Table A3: the descriptive statistics for each item at T1. Table A4: the descriptive statistics for each item at T2. Table A5: correlation matrix of the Wenchuan cross-lagged panel network. Table A6: correlation matrix of the Ya'an cross-lagged panel network. Table A7: ranking of centrality indices for dysphoria symptoms in two samples.

Supplementary 2 Figure A1: autoregressive edge weights (standardized) for each symptom in two networks.

Supplementary 3 Figure A2: 95% CIs of each edge in two networks.

Supplementary 4 Figure A3: stability of BEI for two samples.

Supplementary 5 Figure A4: bootstrapped BEI difference tests in two samples.

Supplementary 6 Figure A5: bootstrapped difference tests between edge weights of the two samples.
