# Supplementary material for: Comorbidity Patterns of Posttraumatic Stress Disorder and Depression Symptoms: Cross-Validation in Two Postearthquake Child and Adolescent Samples
Source: Depress Anxiety. 2023 Nov 8;2023:4453663. doi: 10.1155/2023/4453663 (PMC11921845; doi:10.1155/2023/4453663)
Supplement: Supplementary 1 — Table A1: prevalence and comorbidity of PTSD and depression in two samples. Table A2: the labels and abbreviations for each CPSS and CES-DC item. Table A3: the descriptive statistics for each item at T1. Table A4: the descriptive statistics for each item at T2. Table A5: correlation matrix of the Wenchuan cross-lagged panel network. Table A6: correlation matrix of the Ya'an cross-lagged panel network. Table A7: ranking of centrality indices for dysphoria symptoms in two samples. [file 4453663.f1.zip › Supplementary-20231019.docx]

**Supplementary** **material**

**Table A1,** Prevalence and comorbidity of PTSD and depression in two samples.

**Table A2,** The labels and abbreviations for each CPSS and CES-DC item.

**Table A3,** The descriptive statistics for each item at T1.

**Table A4,** The descriptive statistics for each item at T2.

**Table A5,** Correlation matrix of the Wenchuan cross-lagged panel network.

**Table A6,** Correlation matrix of the Ya'an cross-lagged panel network.

**Table A7,** Ranking of centrality indices for dysphoria symptoms in two sample.

**Figure A1,** Autoregressive edges weights(standardized) for each symptom in two network.

**Figure A2,** 95% CIs of each edge in two network.

**Figure A3,** Stability of BEI for two sample.

**Figure A4,** Bootstrapped BEI difference tests in two sample.

**Figure A5,** Bootstrapped difference tests between edge-weights of the two samples.

Table A1

*Prevalence and comorbidity of PTSD and depression in two samples*

| **Sample** | **PTSD** | **Depression** | **Comorbidity** |
| --- | --- | --- | --- |
| Wenchuan-T1 | 795（52.8%） | 825（54.8%） | 599（39.8%） |
| Wenchuan-T2 | 724（48.1%） | 807（53.6%） | 553（36.7%） |
| Yaan-T1 | 402（55.8%） | 473（65.7%） | 345（47.9%） |
| Yaan-T2 | 354（49.2%） | 458（63.6%） | 307（42.6%） |

Table A2

*The labels and abbreviations for each CPSS and CES-DC item*

| **Labels** | | **Abbreviations** | **Measures** | **Note** |
| --- | --- | --- | --- | --- |
| B1 | Traumatic dreams | | CPSS/PCL-5 |  |
| B2 | Intrusive memories, thoughts, or images of the trauma | | CPSS/PCL-5 |  |
| B3 | Upset (at reminders of the trauma) | | CPSS/PCL-5 |  |
| B4 | Flashbacks | | CPSS/PCL-5 |  |
| B5 | Physiological reactivity | | CPSS/PCL-5 |  |
| C1 | Loss of interest | | CPSS/PCL-5 | Dysphoria Symptoms |
| C2 | Future foreshortening | | CPSS/PCL-5 | Dysphoria Symptoms |
| C3 | Avoid activities reminiscent of the trauma | | CPSS/PCL-5 |  |
| C4 | Emotionally numb | | CPSS/PCL-5 | Dysphoria Symptoms |
| C5 | Difficulty remembering important aspects of the trauma | | CPSS/PCL-5 | Dysphoria Symptoms |
| C6 | Feeling distant or cut off from others | | CPSS/PCL-5 | Dysphoria Symptoms |
| C7 | Avoid thoughts and feelings about the trauma | | CPSS/PCL-5 |  |
| D3 | Exaggerated startle | | CPSS/PCL-5 |  |
| D4 | Hyper-vigilant | | CPSS/PCL-5 |  |
| D5 | Irritable behavior | | CPSS/PCL-5 | Dysphoria Symptoms |
| A1 | Bothered by things that usually don’t bother me | | CES-DC |  |
| A2 | Did not feel like eating | | CES-DC |  |
| A3 | Could not shake off the blues even with help from my family or friends | | CES-DC |  |
| A6 | I felt depressed | | CES-DC |  |
| A7 | Too tired to do anything | | CES-DC |  |
| A9 | Feeling that everything i did was useless | | CES-DC |  |
| A10 | I felt fearful | | CES-DC |  |
| A13 | I talked less than usual | | CES-DC |  |
| A14 | I felt lonely | | CES-DC |  |
| A15 | People were unfriendly | | CES-DC |  |
| A17 | I had crying spells | | CES-DC |  |
| A18 | I felt sad | | CES-DC |  |
| A19 | I felt that people dislike me | | CES-DC |  |
| A20 | I could not get “going” | | CES-DC |  |
| A4 | Good as other kids | | CES-DC | [Positive](javascript:;) Symptoms |
| A8 | Something good going to happen | | CES-DC | [Positive](javascript:;) Symptoms |
| A12 | Was happy | | CES-DC | [Positive](javascript:;) Symptoms |
| A16 | Had a good time | | CES-DC | [Positive](javascript:;) Symptoms |

Table A3

*The descriptive statistics for each item at T1*

| **Labels** | **Wenchuan(T1)** | | | |  | **Ya'an(T1)** | | | |  |
| --- | --- | --- | --- | --- | --- | --- | --- | --- | --- | --- |
|  | ***M*** | ***SD*** | ***S*** | ***K*** |  | ***M*** | ***SD*** | ***S*** | ***K*** |  |
| B1 | 0.8 | 0.77 | 0.8 | 0.39 |  | 0.84 | 0.73 | 0.79 | 0.77 |  |
| B2 | 0.97 | 0.85 | 0.63 | -0.17 |  | 0.81 | 0.79 | 0.93 | 0.73 |  |
| B3 | 1.17 | 0.9 | 0.4 | -0.6 |  | 0.89 | 0.86 | 0.8 | 0.05 |  |
| B4 | 0.81 | 0.84 | 0.85 | 0.1 |  | 0.71 | 0.81 | 0.96 | 0.26 |  |
| B5 | 0.69 | 0.81 | 1.05 | 0.51 |  | 0.65 | 0.84 | 1.18 | 0.65 |  |
| C1 | 0.87 | 0.65 | 0.64 | 1.31 |  | 0.85 | 0.72 | 0.71 | 0.66 |  |
| C2 | 0.56 | 0.78 | 1.32 | 1.09 |  | 0.72 | 0.84 | 1.02 | 0.32 |  |
| C3 | 0.79 | 0.83 | 0.85 | 0.07 |  | 0.65 | 0.83 | 1.12 | 0.46 |  |
| C4 | 0.72 | 0.81 | 0.95 | 0.27 |  | 0.63 | 0.82 | 1.24 | 0.94 |  |
| C5 | 0.71 | 0.9 | 1.14 | 0.38 |  | 0.69 | 0.87 | 1.17 | 0.58 |  |
| C6 | 0.73 | 0.86 | 0.99 | 0.22 |  | 0.7 | 0.82 | 1.01 | 0.31 |  |
| C7 | 0.83 | 0.86 | 0.85 | 0.03 |  | 0.8 | 0.94 | 1.03 | 0.11 |  |
| D3 | 0.96 | 0.85 | 0.66 | -0.13 |  | 0.9 | 0.87 | 0.72 | -0.17 |  |
| D4 | 0.81 | 0.87 | 0.88 | 0.03 |  | 0.87 | 0.9 | 0.8 | -0.2 |  |
| D5 | 0.98 | 0.91 | 0.61 | -0.47 |  | 1.11 | 0.91 | 0.5 | -0.54 |  |
| A1 | 0.81 | 0.73 | 0.69 | 0.37 |  | 0.83 | 0.75 | 0.6 | -0.02 |  |
| A2 | 0.86 | 0.86 | 0.79 | -0.03 |  | 0.83 | 0.84 | 0.7 | -0.3 |  |
| A3 | 0.54 | 0.76 | 1.41 | 1.59 |  | 0.46 | 0.71 | 1.59 | 2.18 |  |
| A6 | 0.93 | 0.86 | 0.68 | -0.21 |  | 0.93 | 0.86 | 0.75 | -0.04 |  |
| A7 | 0.86 | 0.89 | 0.79 | -0.23 |  | 0.85 | 0.93 | 0.86 | -0.21 |  |
| A9 | 0.77 | 0.84 | 0.9 | 0.16 |  | 0.84 | 0.87 | 0.88 | 0.11 |  |
| A10 | 0.78 | 0.9 | 0.94 | -0.05 |  | 0.76 | 0.91 | 1.05 | 0.24 |  |
| A13 | 1.12 | 0.94 | 0.38 | -0.83 |  | 1.22 | 0.98 | 0.32 | -0.93 |  |
| A14 | 0.79 | 0.93 | 0.96 | -0.09 |  | 0.77 | 0.96 | 1.04 | -0.03 |  |
| A15 | 0.68 | 0.87 | 1.17 | 0.53 |  | 0.75 | 0.9 | 1.06 | 0.24 |  |
| A17 | 0.89 | 0.92 | 0.82 | -0.19 |  | 0.86 | 0.92 | 0.89 | -0.05 |  |
| A18 | 0.92 | 0.9 | 0.74 | -0.22 |  | 0.87 | 0.91 | 0.86 | -0.06 |  |
| A19 | 0.73 | 0.89 | 1.08 | 0.31 |  | 0.8 | 0.9 | 1.01 | 0.25 |  |
| A20 | 0.83 | 0.86 | 0.82 | -0.07 |  | 0.89 | 0.91 | 0.8 | -0.18 |  |
| A4 | 1.48 | 1.08 | 0.05 | -1.26 |  | 1.6 | 1.07 | -0.08 | -1.25 |  |
| A8 | 0.93 | 0.91 | 0.79 | -0.13 |  | 1.04 | 0.9 | 0.63 | -0.33 |  |
| A12 | 1.45 | 0.98 | 0.08 | -1 |  | 1.62 | 0.97 | -0.14 | -0.95 |  |
| A16 | 1.53 | 1.02 | -0.03 | -1.13 |  | 1.76 | 1 | -0.25 | -1.05 |  |

Note. *S*: skewness; *K*: kurtosis

Table A4.

*The descriptive statistics for each item at T2*

| **Labels** | **Wenchuan(T2)** | | | |  | **Ya'an(T2)** | | | |  |
| --- | --- | --- | --- | --- | --- | --- | --- | --- | --- | --- |
|  | ***M*** | ***SD*** | ***S*** | ***K*** |  | ***M*** | ***SD*** | ***S*** | ***K*** |  |
| B1 | 0.77 | 0.71 | 0.77 | 0.68 |  | 0.87 | 0.75 | 0.79 | 0.68 |  |
| B2 | 0.76 | 0.79 | 0.92 | 0.47 |  | 0.66 | 0.75 | 1.04 | 0.84 |  |
| B3 | 0.96 | 0.87 | 0.6 | -0.37 |  | 0.7 | 0.79 | 1.04 | 0.64 |  |
| B4 | 0.63 | 0.75 | 1.1 | 0.86 |  | 0.61 | 0.8 | 1.25 | 0.94 |  |
| B5 | 0.54 | 0.75 | 1.34 | 1.31 |  | 0.62 | 0.82 | 1.2 | 0.66 |  |
| C1 | 0.82 | 0.67 | 0.71 | 1.07 |  | 0.82 | 0.78 | 0.82 | 0.46 |  |
| C2 | 0.54 | 0.77 | 1.41 | 1.44 |  | 0.76 | 0.89 | 1.02 | 0.21 |  |
| C3 | 0.71 | 0.8 | 1.03 | 0.59 |  | 0.52 | 0.74 | 1.48 | 1.95 |  |
| C4 | 0.67 | 0.81 | 1.09 | 0.56 |  | 0.6 | 0.79 | 1.24 | 0.96 |  |
| C5 | 0.65 | 0.8 | 1.12 | 0.68 |  | 0.61 | 0.82 | 1.31 | 1.1 |  |
| C6 | 0.7 | 0.86 | 1.08 | 0.33 |  | 0.73 | 0.86 | 0.95 | 0.04 |  |
| C7 | 0.77 | 0.84 | 0.98 | 0.39 |  | 0.65 | 0.83 | 1.3 | 1.19 |  |
| D3 | 0.92 | 0.82 | 0.69 | 0 |  | 0.92 | 0.87 | 0.72 | -0.14 |  |
| D4 | 0.73 | 0.81 | 0.95 | 0.33 |  | 0.82 | 0.88 | 0.89 | 0.07 |  |
| D5 | 0.97 | 0.89 | 0.64 | -0.34 |  | 1.08 | 0.94 | 0.5 | -0.66 |  |
| A1 | 0.76 | 0.76 | 0.85 | 0.46 |  | 0.78 | 0.76 | 0.91 | 0.8 |  |
| A2 | 0.78 | 0.82 | 0.9 | 0.27 |  | 0.81 | 0.82 | 0.87 | 0.27 |  |
| A3 | 0.5 | 0.72 | 1.4 | 1.55 |  | 0.44 | 0.72 | 1.66 | 2.27 |  |
| A6 | 0.86 | 0.83 | 0.75 | -0.01 |  | 0.88 | 0.82 | 0.77 | 0.14 |  |
| A7 | 0.81 | 0.87 | 0.87 | 0.02 |  | 0.84 | 0.86 | 0.78 | -0.14 |  |
| A9 | 0.77 | 0.81 | 0.82 | 0.04 |  | 0.83 | 0.87 | 0.82 | -0.12 |  |
| A10 | 0.73 | 0.86 | 1.03 | 0.3 |  | 0.69 | 0.87 | 1.21 | 0.75 |  |
| A13 | 1.11 | 0.95 | 0.46 | -0.72 |  | 1.11 | 0.94 | 0.46 | -0.72 |  |
| A14 | 0.74 | 0.91 | 1.08 | 0.27 |  | 0.84 | 0.95 | 0.92 | -0.17 |  |
| A15 | 0.69 | 0.85 | 1.11 | 0.51 |  | 0.8 | 0.93 | 0.97 | -0.05 |  |
| A17 | 0.81 | 0.9 | 0.93 | 0.02 |  | 0.82 | 0.92 | 0.94 | -0.03 |  |
| A18 | 0.83 | 0.88 | 0.89 | 0.04 |  | 0.83 | 0.9 | 0.89 | -0.01 |  |
| A19 | 0.72 | 0.86 | 1.09 | 0.46 |  | 0.85 | 0.92 | 0.94 | 0.08 |  |
| A20 | 0.82 | 0.85 | 0.84 | 0.02 |  | 0.86 | 0.91 | 0.87 | -0.08 |  |
| A4 | 1.52 | 1.05 | -0.02 | -1.21 |  | 1.47 | 1.07 | 0.03 | -1.26 |  |
| A8 | 0.92 | 0.86 | 0.73 | -0.09 |  | 1.06 | 0.95 | 0.64 | -0.49 |  |
| A12 | 1.52 | 0.96 | 0.04 | -0.96 |  | 1.51 | 1.01 | 0.01 | -1.11 |  |
| A16 | 1.6 | 1 | -0.1 | -1.06 |  | 1.64 | 1.03 | -0.11 | -1.14 |  |

*Note. S:* skewness; K: kurtosis

Table A5

*Correlation matrix of the Wenchuan cross-lagged panel network*

*
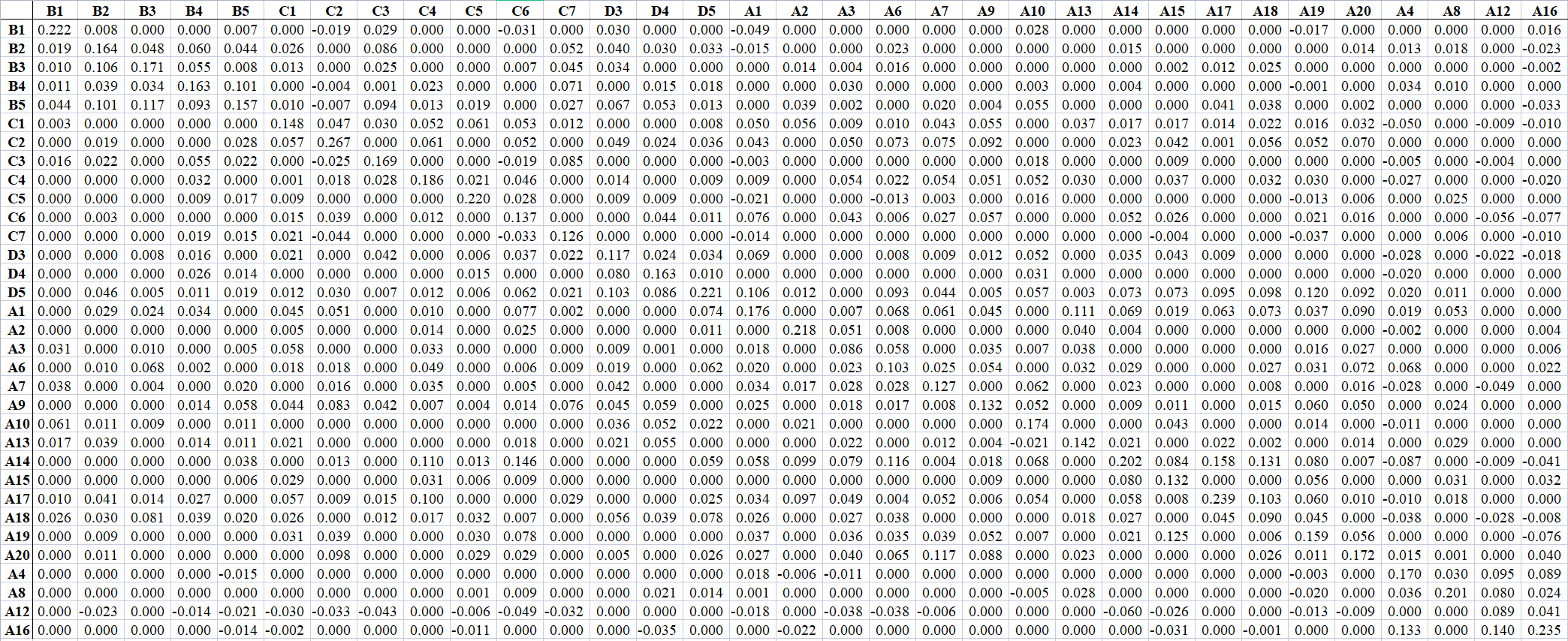
Note.* Independent variables (i.e., predictors) are in rows, and dependent variables are in columns. Autoregressive edges are presented along the diagonal. Node abbreviations are provided in *Table A2*.

Table A6

*Correlation matrix of the* *Ya'an cross-lagged panel network*

*Note.*
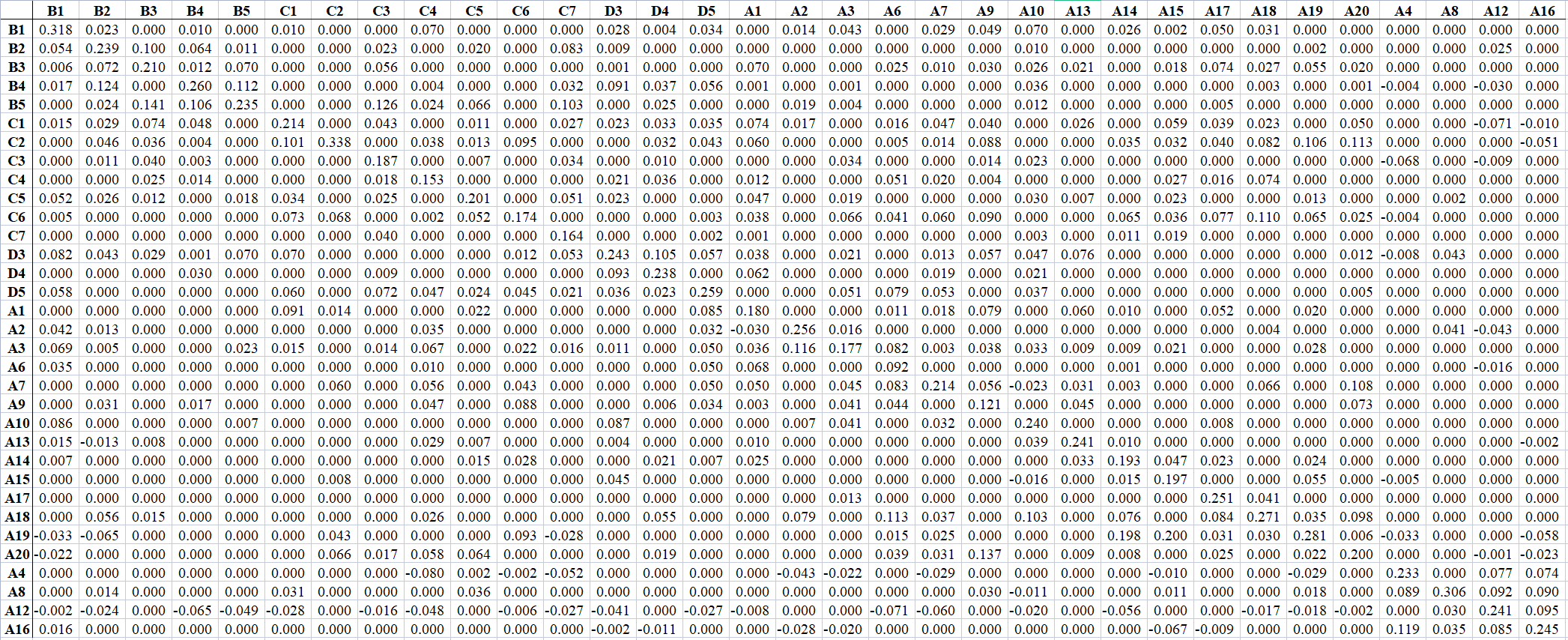
Independent variables (i.e., predictors) are in rows, and dependent variables are in columns. Autoregressive edges are presented along the diagonal. Node abbreviations are provided in *Table A2 .*

Table A7

*Ranking of centrality indices for dysphoria symptoms in two sample*

| **Node** | **Wenchuan** | **Ya'an** |
| --- | --- | --- |
| **D5** | **1** | 8 |
| **C6** | 13 | **1** |
| **C2** | **2** | **2** |
| **C1** | 9 | 5 |
| **C4** | 8 | 12 |
| **C5** | 28 | 16 |


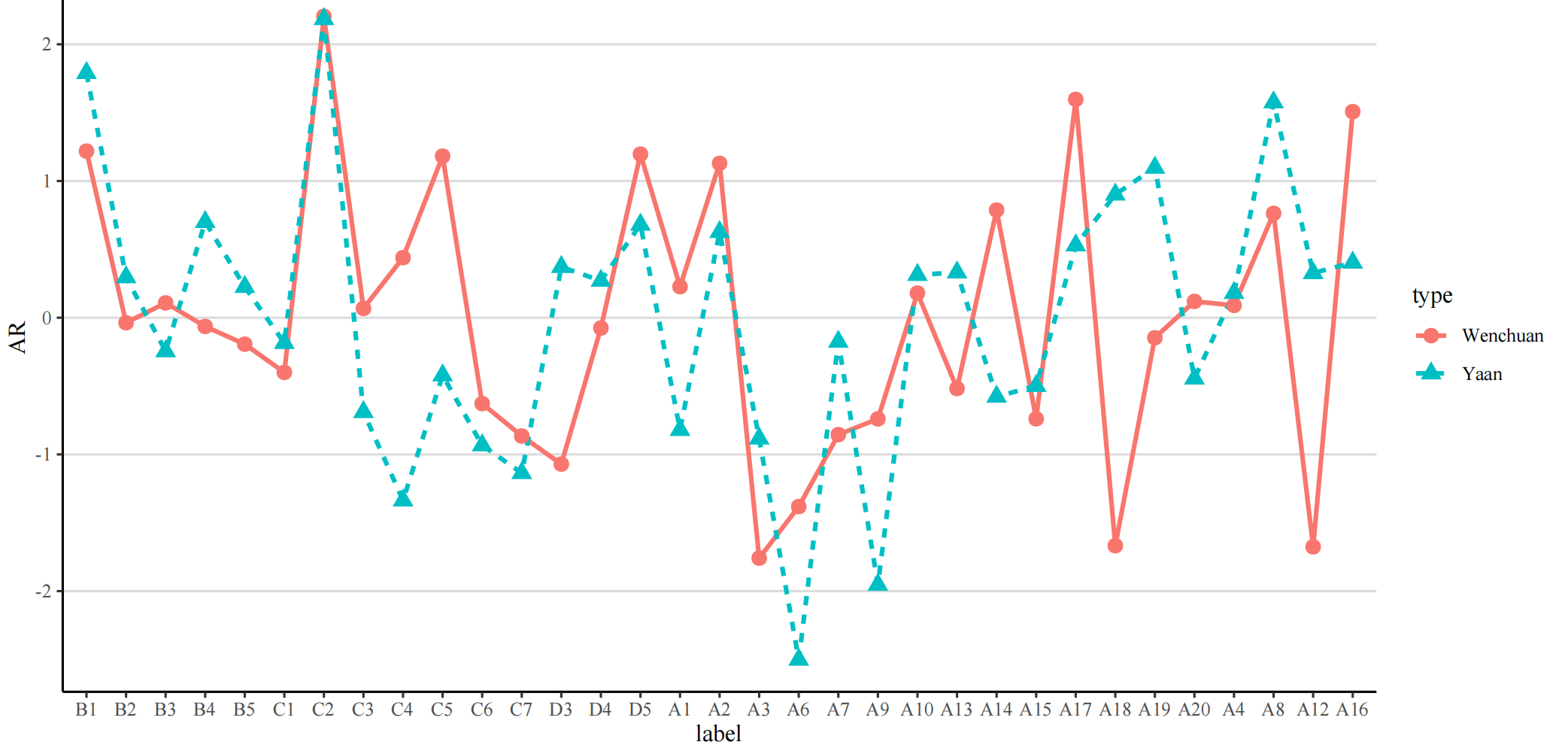
Figure A1

*Autoregressive edges weights(standardized) for each symptom in two network. See Table A3 for items corresponding to each node.*


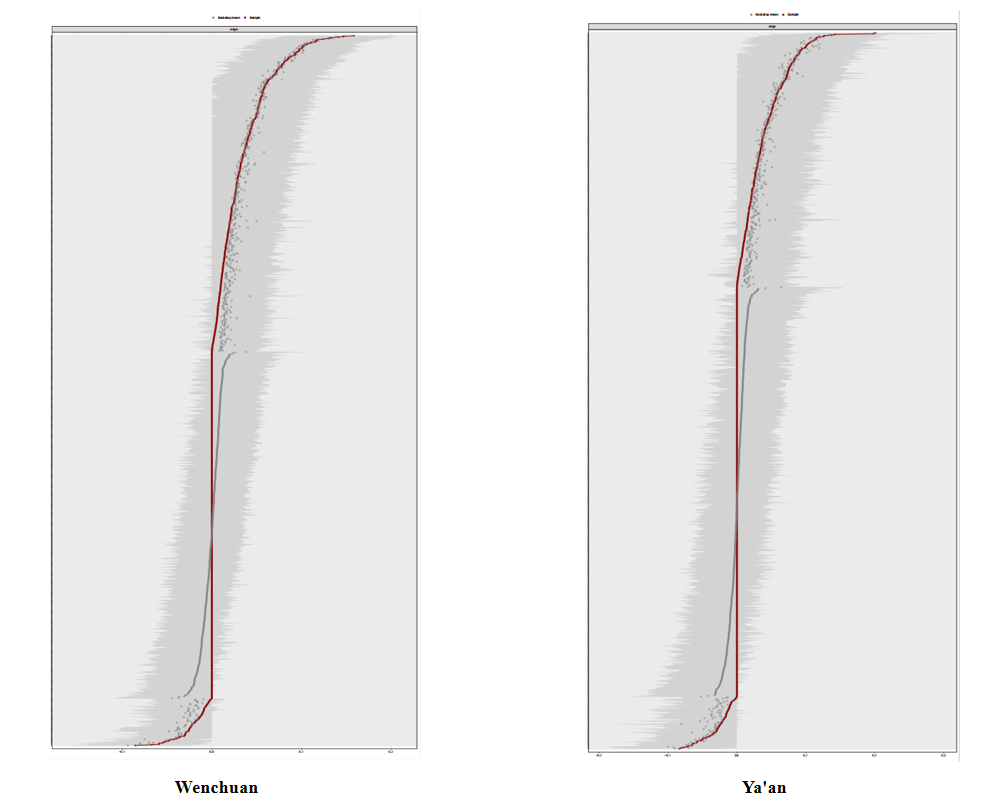
Figure A2.

*95% CIs of each edge in two network.*


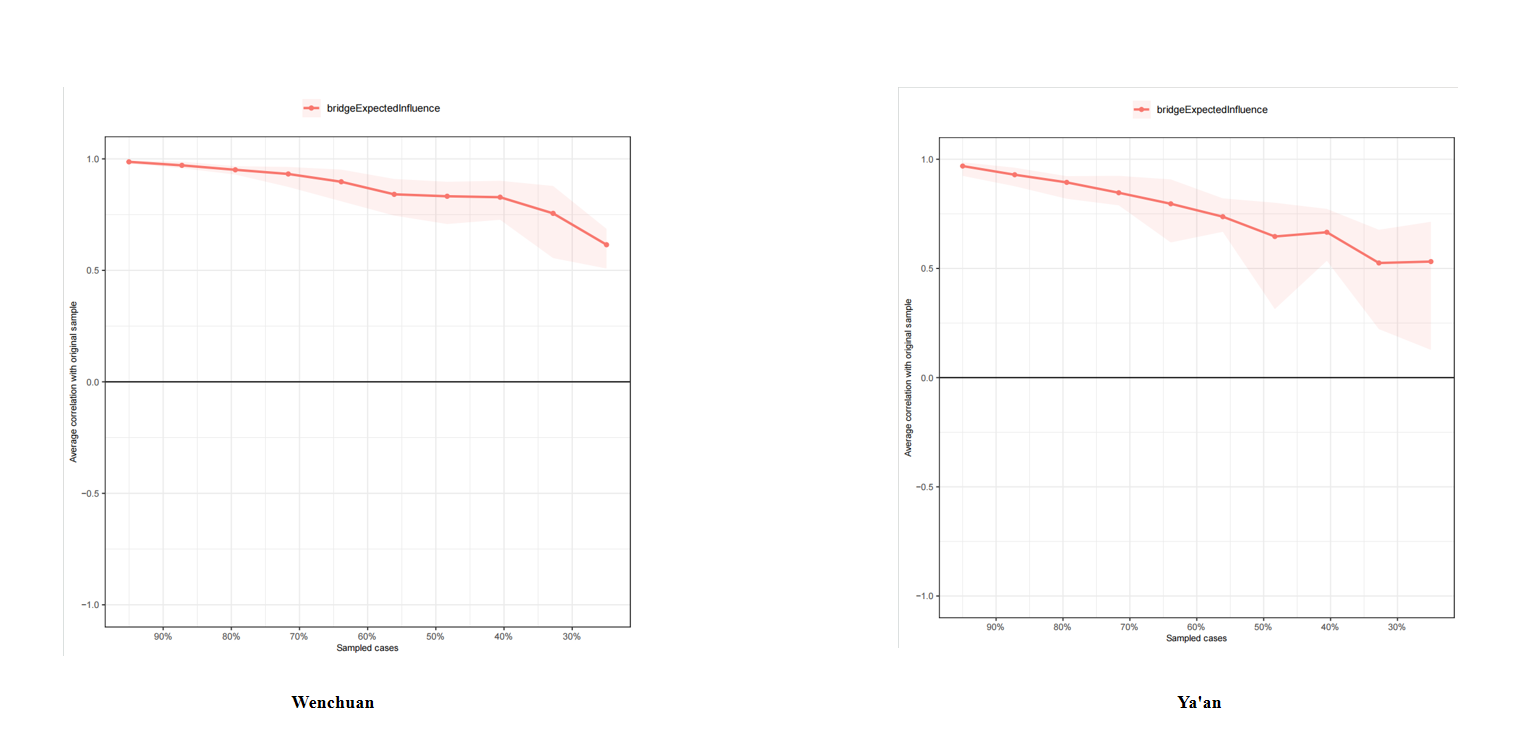


Figure A3.

*Stability of BEI for two sample*


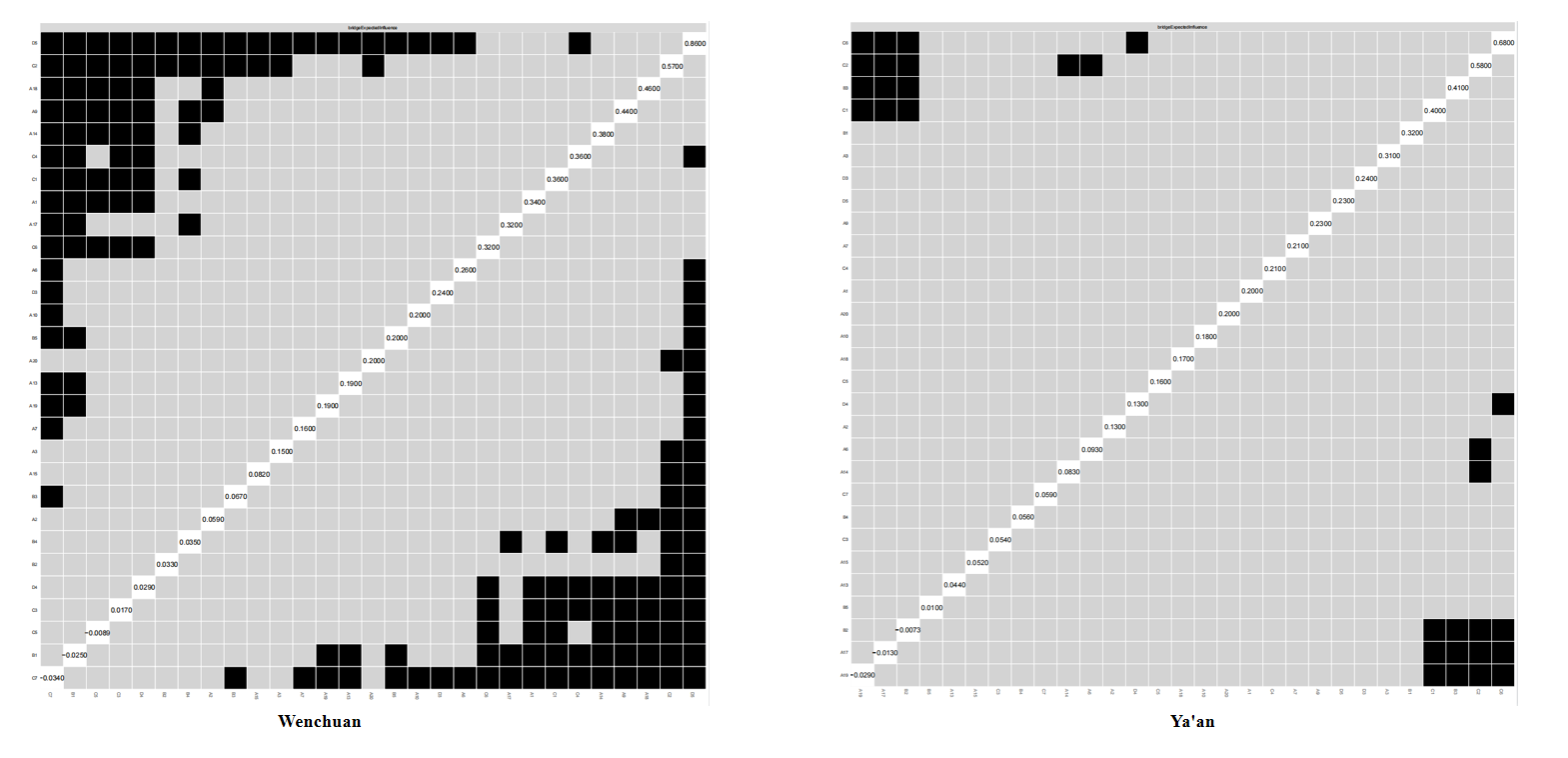
Figure A4.

*Bootstrapped BEI difference tests in two sample*

*
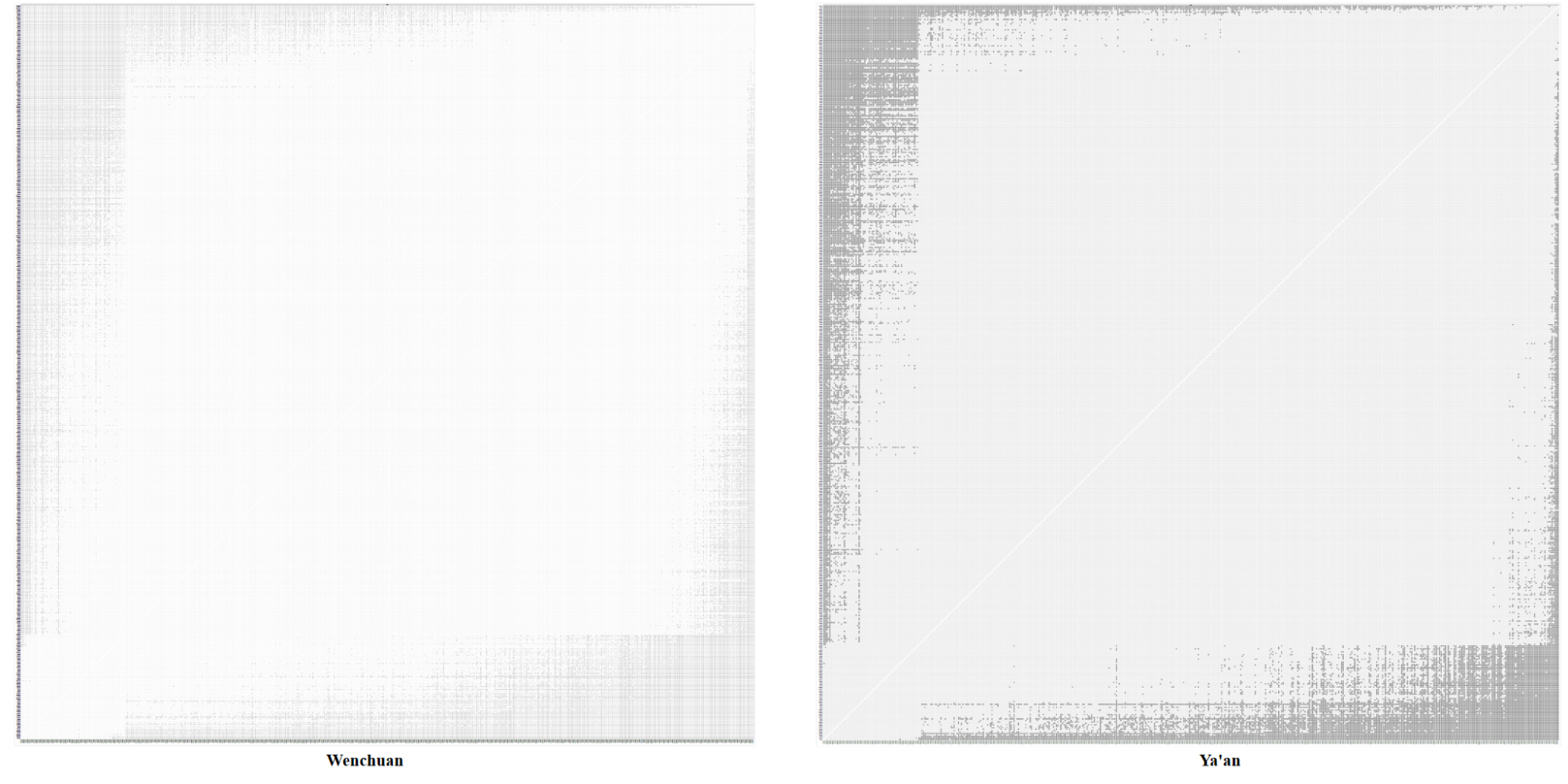
*

Figure A5.

*Bootstrapped difference tests between edge-weights of the two samples*
