# Supplementary figures and images for: Comorbidity Patterns of Posttraumatic Stress Disorder and Depression Symptoms: Cross-Validation in Two Postearthquake Child and Adolescent Samples
Source: Depress Anxiety. 2023 Nov 8;2023:4453663. doi: 10.1155/2023/4453663 (PMC11921845; doi:10.1155/2023/4453663)

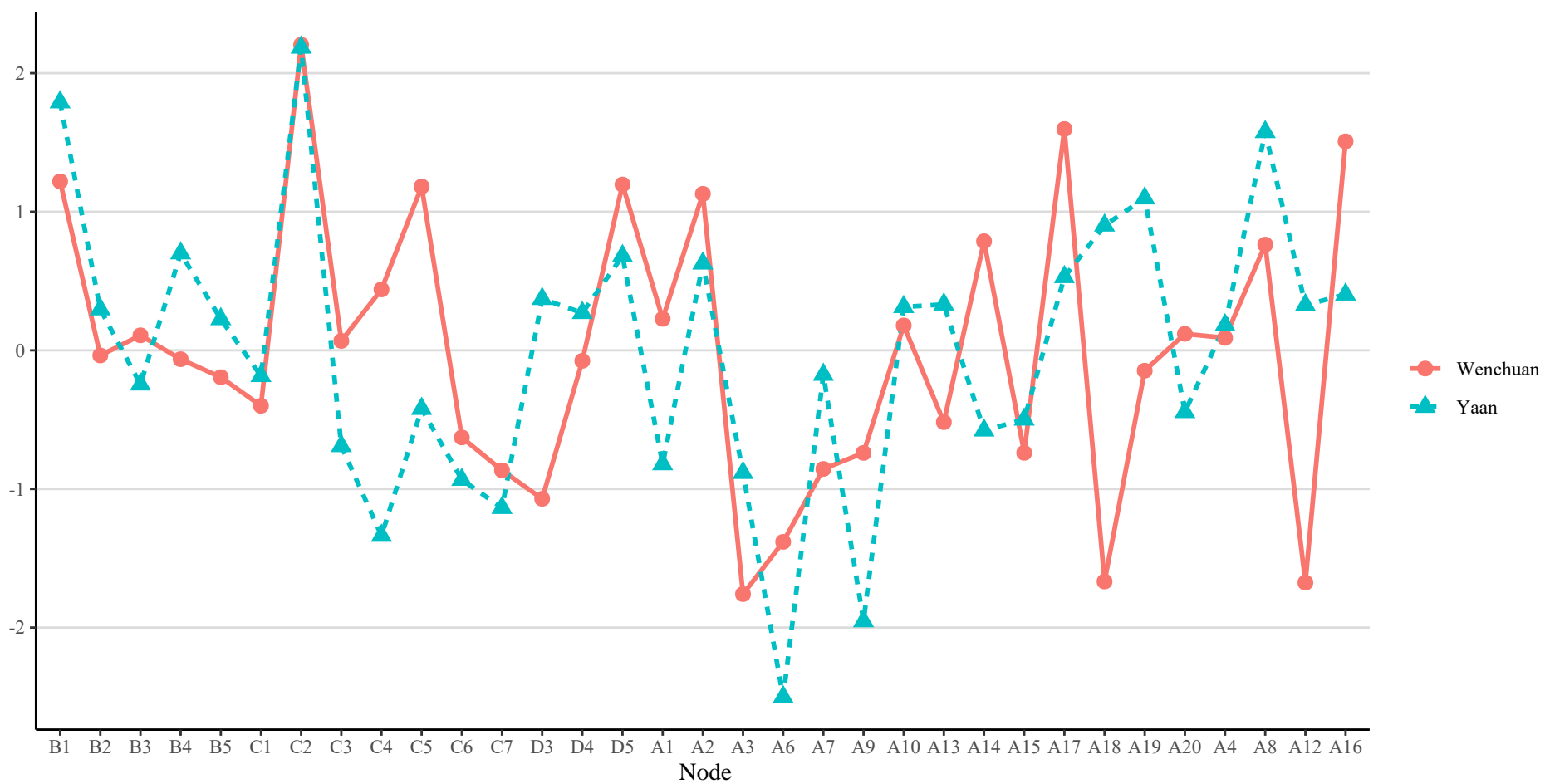

Supplement: Supplementary 2 — Figure A1: autoregressive edge weights (standardized) for each symptom in two networks. [file 4453663.f2.pdf]

• Bootstrap mean • Sample

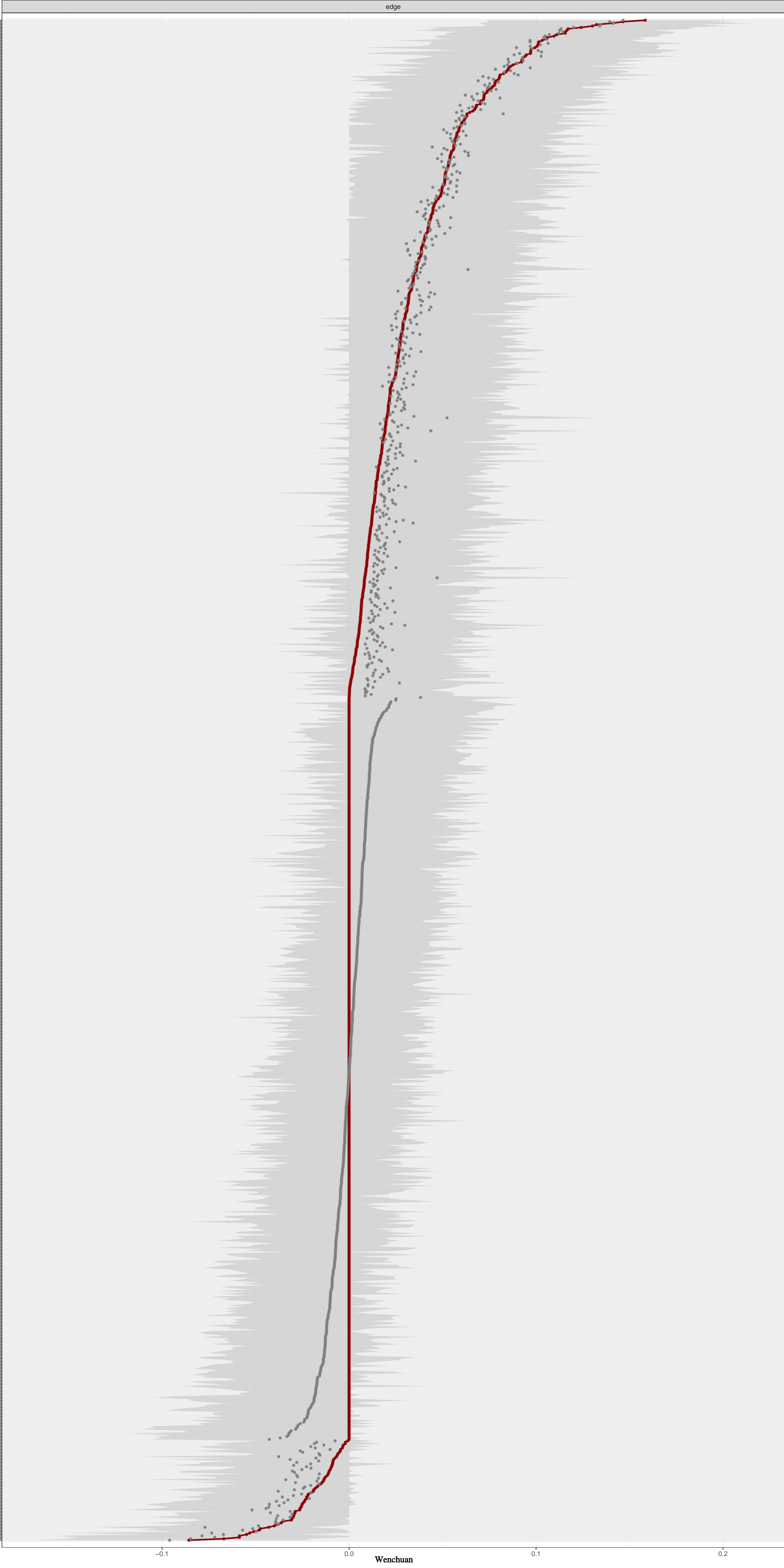

Supplement: Supplementary 3 — Figure A2: 95% CIs of each edge in two networks. [file 4453663.f3.zip › Supplementary-Figure A2-Wenchuan.pdf]

• Bootstrap mean • Sample

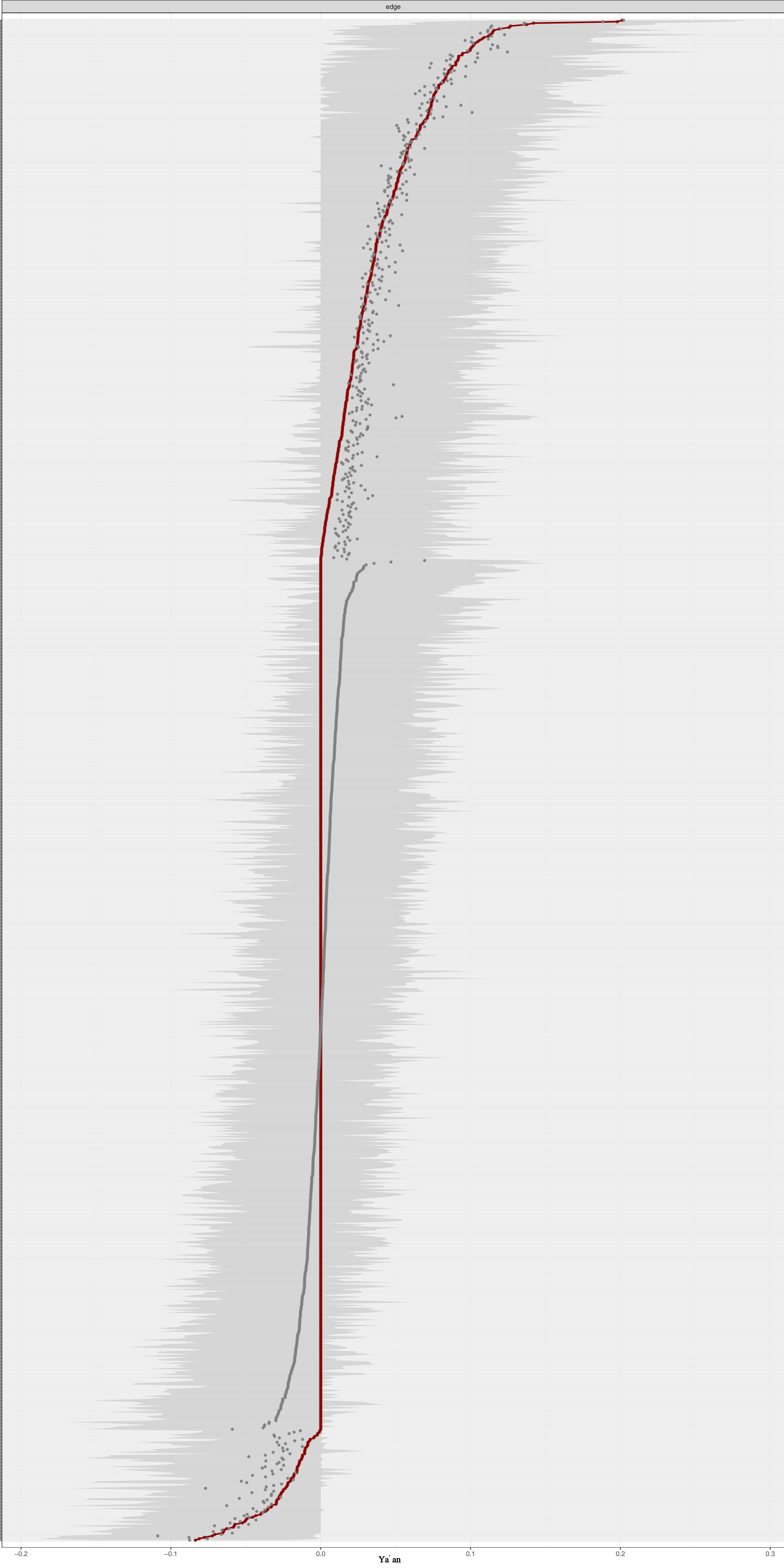

Supplement: Supplementary 3 — Figure A2: 95% CIs of each edge in two networks. [file 4453663.f3.zip › Supplementary-Figure A2-Yaan.pdf]

bridgeExpectedInfluence

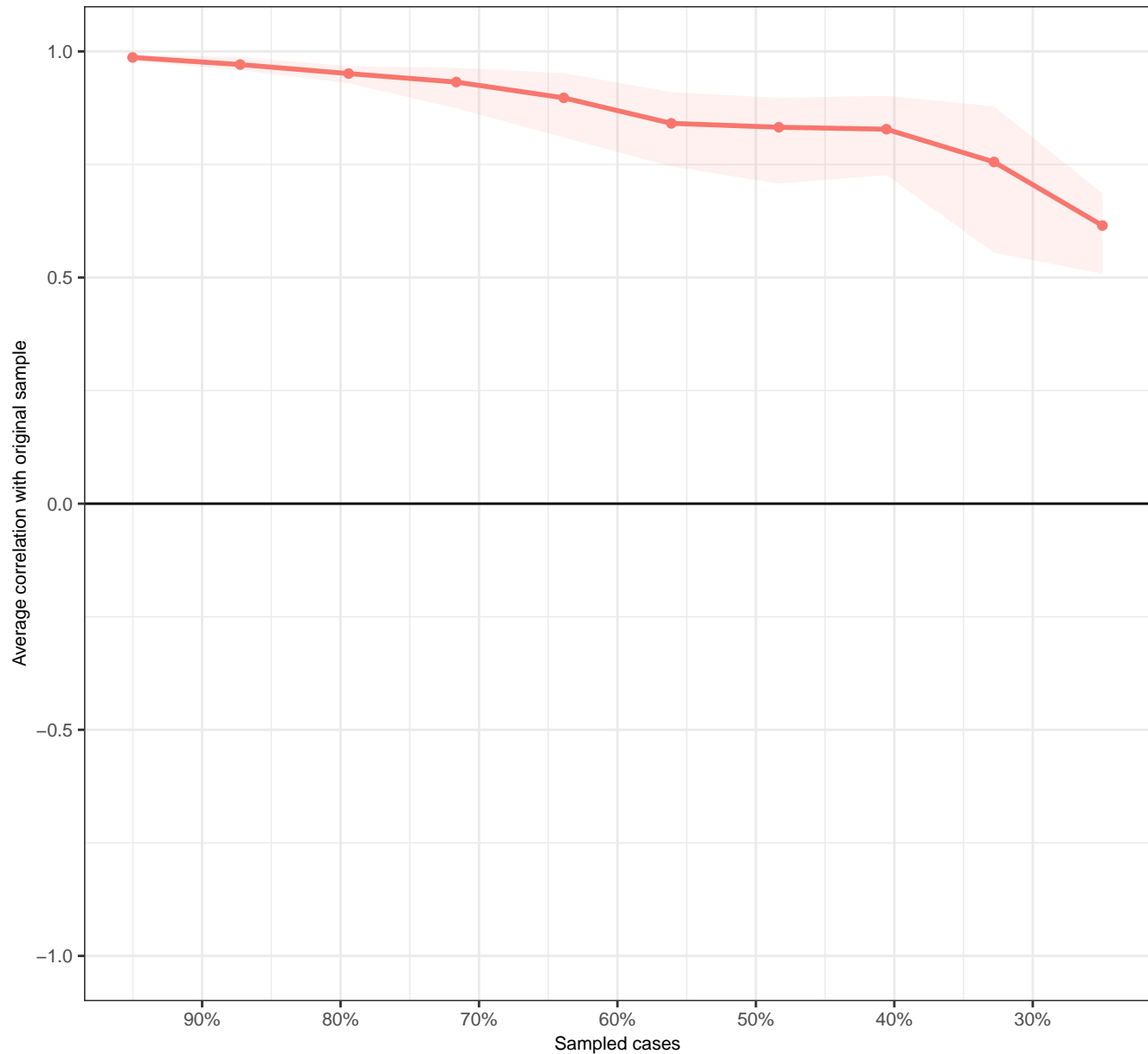

Supplement: Supplementary 4 — Figure A3: stability of BEI for two samples. [file 4453663.f4.zip › Supplementary-Figure A3-Wen.pdf]

bridgeExpectedInfluence

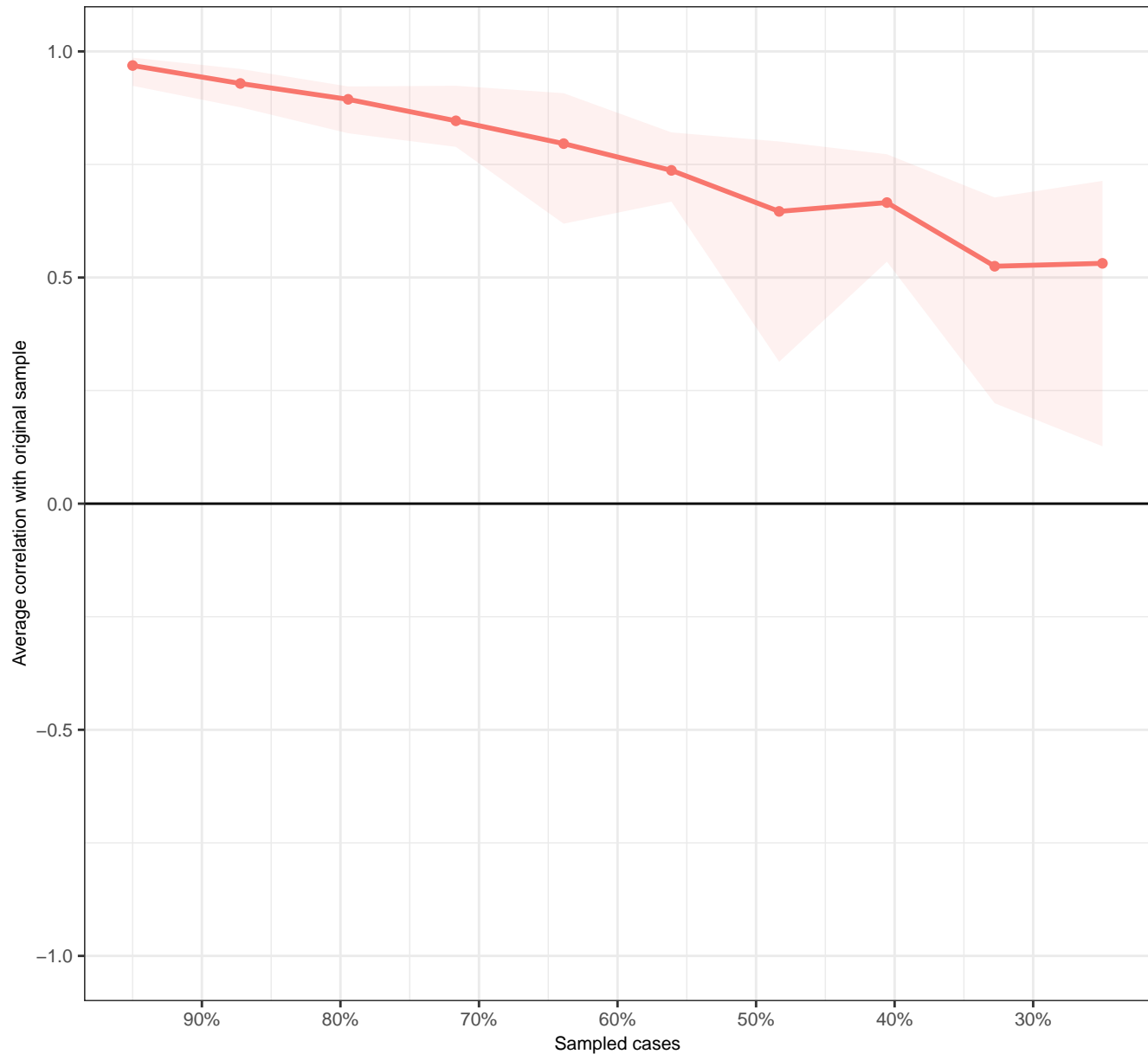

Supplement: Supplementary 4 — Figure A3: stability of BEI for two samples. [file 4453663.f4.zip › Supplementary-Figure A3-Yaan.pdf]

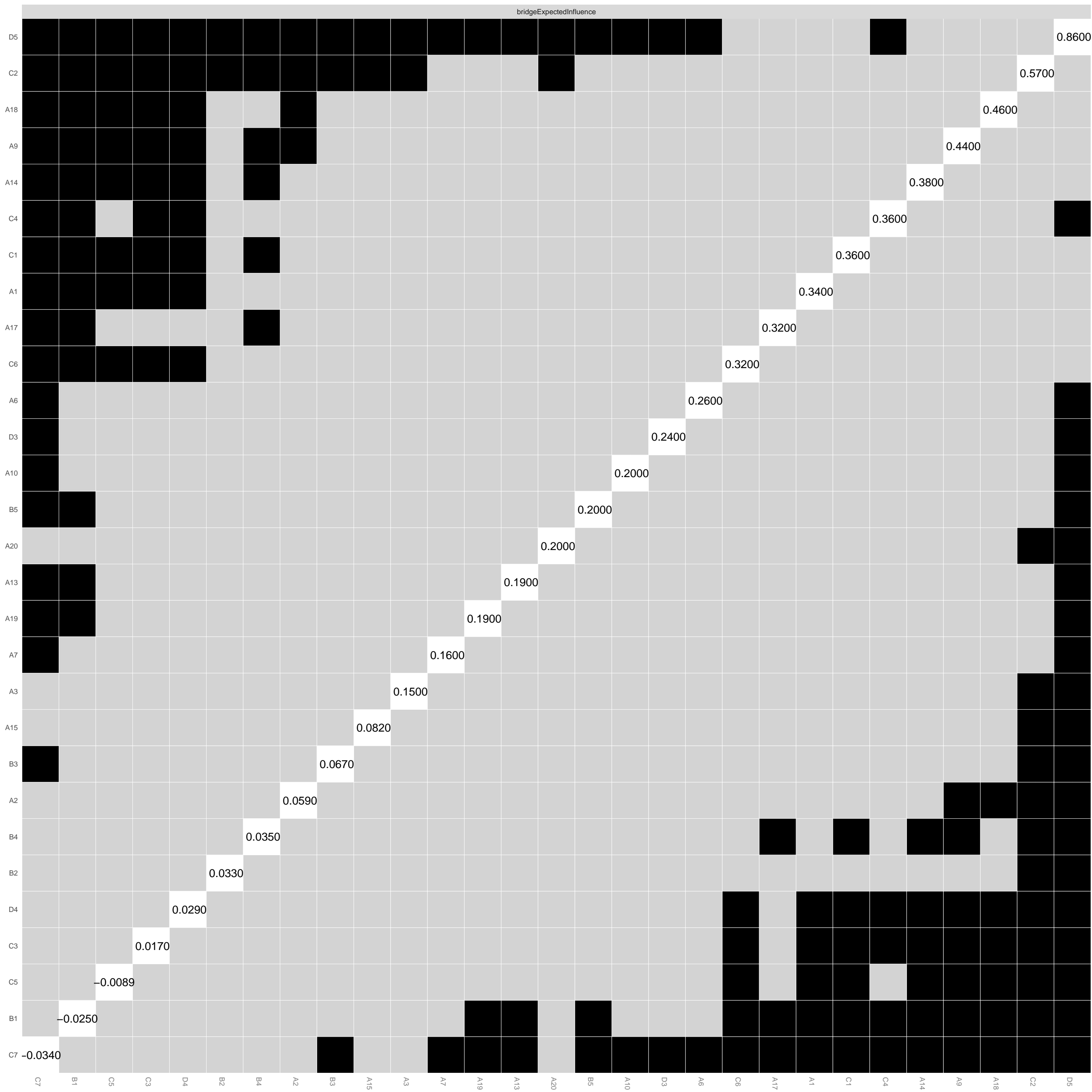

Supplement: Supplementary 5 — Figure A4: bootstrapped BEI difference tests in two samples. [file 4453663.f5.zip › Supplementary-Figure A4-Wenchuan.pdf]
