# Supplementary material for: Comorbidity Patterns of Posttraumatic Stress Disorder and Depression Symptoms: Cross-Validation in Two Postearthquake Child and Adolescent Samples
Source: Depress Anxiety. 2023 Nov 8;2023:4453663. doi: 10.1155/2023/4453663 (PMC11921845; doi:10.1155/2023/4453663)
Supplement: Supplementary 5 — Figure A4: bootstrapped BEI difference tests in two samples. [file Supplementary--Figure A4-Yaan.pdf › Supplementary--Figure A4-Yaan.pdf]

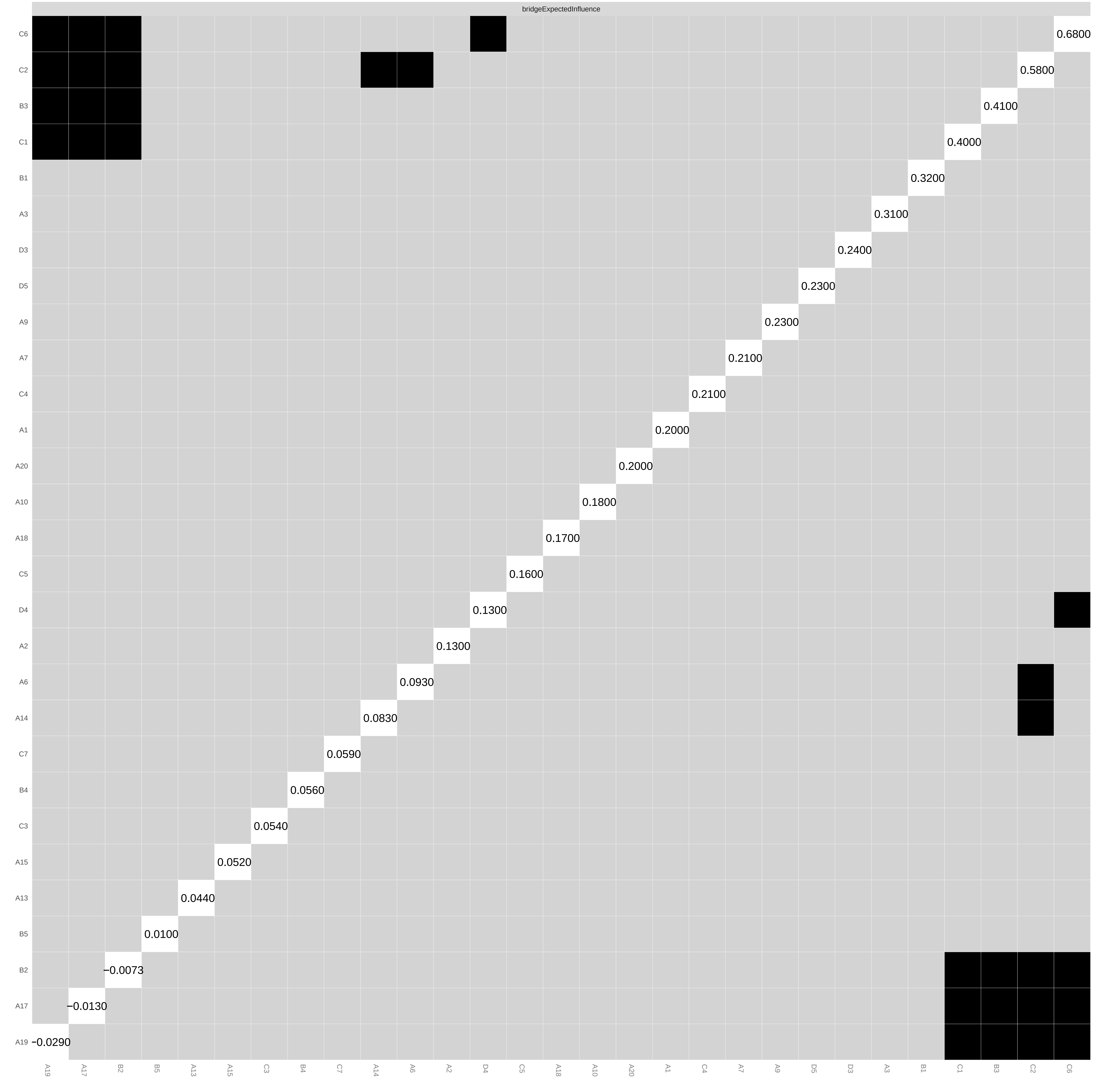

bridgeExpectedInfluence
C6
C2
B3
C1
B1
A3
D3
D5
A9
A7
C4
A1
A20
A10
A18
C5
D4
A2
A6
A14
C7
B4
C3
A15
A13
B5
B2
A17
A19
0.6800
0.5800
0.4100
0.4000
0.3200
0.3100
0.2400
0.2300
0.2300
0.2100
0.2100
0.2000
0.2000
0.1800
0.1700
0.1600
0.1300
0.1300
0.0930
0.0830
0.0590
0.0560
0.0540
0.0520
0.0440
0.0100
−0.0073
−0.0130
−0.0290
A19
A17
B2
B5
A13
A15
C3
B4
C7
A14
A6
A2
D4
C5
A18
A10
A20
A1
C4
A7
A9
D5
D3
A3
B1
C1
B3
C2
C6
