# Supplementary material for: Comorbidity Patterns of Posttraumatic Stress Disorder and Depression Symptoms: Cross-Validation in Two Postearthquake Child and Adolescent Samples
Source: Depress Anxiety. 2023 Nov 8;2023:4453663. doi: 10.1155/2023/4453663 (PMC11921845; doi:10.1155/2023/4453663)

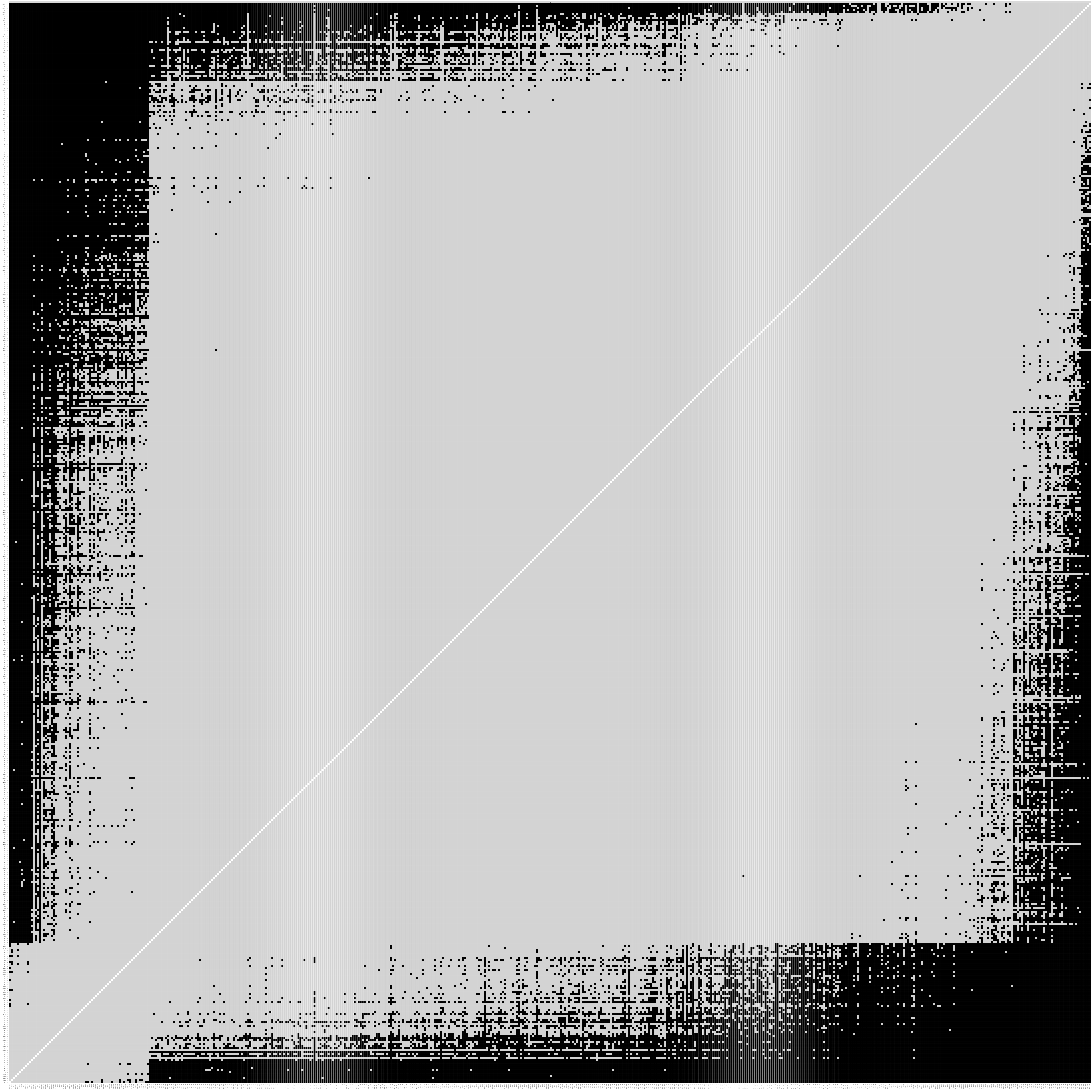

Supplement: Supplementary 6 — Figure A5: bootstrapped difference tests between edge weights of the two samples. [file Supplementary-Figure A5-Wenchuan.pdf › Supplementary-Figure A5-Wenchuan.pdf]
